# Understanding testicular single cell transcriptional atlas: from developmental complications to male infertility

**DOI:** 10.3389/fendo.2024.1394812

**Published:** 2024-07-11

**Authors:** Munichandra Babu Tirumalasetty, Indrashis Bhattacharya, Mohammad Sarif Mohiuddin, Vijaya Bhaskar Baki, Mayank Choubey

**Affiliations:** ^1^ Department of Foundations of Medicine, NYU Grossman Long Island School of Medicine, Mineola, NY, United States; ^2^ Department of Zoology, School of Biological Sciences, Central University of Kerala, Kasargod, Kerala, India; ^3^ Division of Biomedical Sciences, School of Medicine, University of California, Riverside, Riverside, CA, United States

**Keywords:** single-cell RNA-sequencing (Sc-RNA-seq), testis, spatial transcriptomics, spermatogenesis, male infertility

## Abstract

Spermatogenesis is a multi-step biological process where mitotically active diploid (2n) spermatogonia differentiate into haploid (n) spermatozoa via regulated meiotic programming. The alarming rise in male infertility has become a global concern during the past decade thereby demanding an extensive profiling of testicular gene expression. Advancements in Next-Generation Sequencing (NGS) technologies have revolutionized our empathy towards complex biological events including spermatogenesis. However, despite multiple attempts made in the past to reveal the testicular transcriptional signature(s) either with bulk tissues or at the single-cell, level, comprehensive reviews on testicular transcriptomics and associated disorders are limited. Notably, technologies explicating the genome-wide gene expression patterns during various stages of spermatogenic progression provide the dynamic molecular landscape of testicular transcription. Our review discusses the advantages of single-cell RNA-sequencing (Sc-RNA-seq) over bulk RNA-seq concerning testicular tissues. Additionally, we highlight the cellular heterogeneity, spatial transcriptomics, dynamic gene expression and cell-to-cell interactions with distinct cell populations within the testes including germ cells (Gc), Sertoli cells (Sc), Peritubular cells (PTc), Leydig cells (Lc), etc. Furthermore, we provide a summary of key finding of single-cell transcriptomic studies that have shed light on developmental mechanisms implicated in testicular disorders and male infertility. These insights emphasize the pivotal roles of Sc-RNA-seq in advancing our knowledge regarding testicular transcriptional landscape and may serve as a potential resource to formulate future clinical interventions for male reproductive health.

## 1 Introduction

During the past two decades, an alarming rise in infertility particularly in developed/developing countries has become a matter of great concern (1). Around 15% of couples suffering from infertility globally, out of which 50%, is exclusively caused by the male partner (2). Male infertility is a multifaceted pathological condition with diverse manifestations, ranging from total absence of testicular sperm to specific changes in sperm quality (3). Chromosomal aberrations (like Klinefelter syndrome 47XXY) or variance (like 9qh+ heteromorphism) and other genetic mutations contribute upto 15-20% of total male infertility; however around 30-50% of cases remain untreatable due to idiopathic in origin/nature (4–6). Therefore, the mechanistic details of testicular spermatogenesis need to be reexamined at the cellular and molecular level (7, 8).

**Figure 1:**
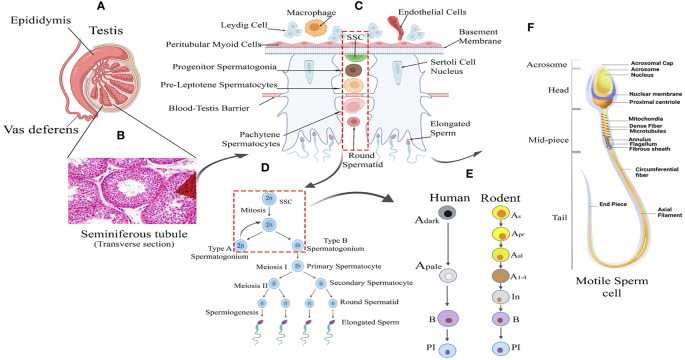
Testicular tissue architecture and Spermatogenic Development. **(A)** Testis Anatomy: Cross-section illustrating the testis with internal cellular composition and associated structures: the epididymis and vas deferens. **(B)** Seminiferous Tubule: Histological image showing the transverse section of a seminiferous tubule where spermatogenesis occurs. **(C)** Spermatogenesis Overview: Schematic representation of spermatogenesis within the seminiferous tubule, from spermatogonial stem cells (SSC) to mature spermatozoa. **(D)** Sperm Maturation: Detailed progression of sperm development from type A spermatogonium through meiosis to form mature sperm. **(E)** Human vs. Rodent Spermatogenesis: Comparative stages of spermatogenesis between humans and rodents, highlighting the differentiation of spermatogonial cells. **(F)** Sperm Cell Morphology: Detailed diagram of a motile sperm cell, showcasing its various structural components from head to tail.

Spermatogenesis is gonadotropin regulated, highly synchronized, developmental programme involving multi-step events like stem cell renewal/differentiation, epigenomic remodeling, and meiotic divisions for generating millions of spermatozoa inside testes (9–11). The continuity of this process throughout adult life is supported by an active spermatogonial stem cell (SSC) population that critically maintains the key balance between the capacity of self-renewal (to sustain the stem cell population) and differentiation [to produce spermatogonial progenitor cells (SPCs), which subsequently develop into mature sperm via meiotic divisions] (12–14). Therefore, the cellular and molecular landscape associated with the SSC micro-environment turns out to be critical for regulating male fertility. The rapid intrinsic speed (around 1000-1500 sperm per heartbeat in adult men) of spermatogenic progression significantly highlights the degree of orchestration governing the transcriptional dynamics of testicular cells (15–17). **Figure 1** represents the histological architecture of the testis showing arrays of multiple developing germ cells (Gc) (from SSC to mature motile sperms) alongside with visual representation of the different stages and cellular interactions essential for spermatogenesis.

Functional and/or anatomical defects in testicular somatic and/or Gc lead to spermatogenic impairment (with a qualitative and quantitative decline in sperm count clinically defined as azoospermia, oligozoospermia, teratozoospermia, and asthenospermia, etc.) causing male sub-infertility/infertility (18). Azoospermia, (the most severe form where semen without sperm) are of two types: obstructive azoospermia (OA) and non-obstructive azoospermia (NOA). OA stems from normal sperm production but obstructed sperm delivery, while NOA, accounting for around 10% of male infertility, results from lack of spermatogenesis in the testis (19). Three specific clinical categories are described in NOA as primary testicular failure like Sertoli cell only syndrome (SCOS)/Gc aplasia, Gc maturational arrest and hypo-spermatogenesis (20, 21). **Supplementary Table 1** highlights (A) clinical categories of semen quality, (B) etiologies and frequencies of male infertility and (C) the hallmarks of primary testicular failure.

Notably, multiple genetic complexities involving over 2,000 spermatogenic genes observed in different forms of male infertility having substantial variations in semen quality and testicular histology (5, 22). Over recent decades assisted reproductive techniques (ART) e.g.- *in vitro* fertilization (IVF) and intra-cytoplasmic sperm injection (ICSI) have successfully facilitated biological fatherhood for men with extremely low sperm counts (3). However, addressing the fundamental basis of poor sperm quality and quantity remains challenging due to a limited understanding of the intricate molecular and cellular interactions driving human sperm production (18, 23).

An impressive progress in deploying high-throughput sequencing technologies in biomedical research has been observed during the past decade (24). Within this scenario, both bulk and single-cell RNA-sequencing (Sc-RNA-seq) collectively have emerged as revolutionary tools, revealing the molecular complexities governing testicular physiology and elucidating mechanisms underlying testicular dysregulation/disorders (25). However, analyzing bulk RNAs from diverse cell types faces adverse challenges in detecting expression data in rare cell populations, possibly diluting the presence of uncommon transcripts. In contrast, Sc-RNA-seq generates distinct RNA fingerprints for each cell (at single-cell resolution) type, transforming our understanding of differential transcriptomic profiles from complex tissues like the testis and therefore found to be critically informative in the field of clinical endocrinology, especially in deciphering infertility (26, 27). **Table 1** summarizes the advantages of the Sc-RNA-seq strategy over the conventional bulk transcriptomic approach.

**Table 1:** Summarizes the essential aspects of different RNA-Seq strategies, including their aims, key features, popular platforms, merits, limitations, and direct applications.

Approach/ Methods	Aim	Protocol	Popular platforms	Merits	Limitations	Direct Application(s)	Key References
**Bulk RNA-** **Seq**	To investigate the global gene expression profile of an organ/tissue without discriminating different cell populations	Total RNAs extracted from the tissues, depletion of rRNAs, cDNA preparation, adaptor ligation, library preparation, amplification, sequencing and generation of FASTQ files	Illumina (HiSeq/ NovaSeq etc), Ion torrent, Pacific Bio- Sciences & Oxford Nanopore etc	Well-developed for both short and long reads, cost-effective high throughput technique	Single cell specific transcriptomic data are unachievable, noisy gene expression profile with limited accuracy and detection rate, no spatial information available	Typically used for evaluating tissue and age specific differential gene expression profiles in model (reference guided genome assembly) and non-model (Trinity based *de novo* assembly) organisms, Identifications of splice variants/ transcript isoforms etc	(24)
**Single Cell RNA-seq**	To investigate the gene transcription at the resolution of a single cell level obtained from a complex tissue/ organ	Single-cell suspension is prepared [either by crude enzymatic digestion /mechanical dissociation, or sorting cells labeled by transgenic reporters (GFP/RFP/YFP etc) or based on antibodies etc.] followed by RNA extraction and subsequent tagging of the target transcripts with cell-specific unique molecular identifiers (UMIs), next cDNA preparation and other protocol same as performed for Bulk RNA-Seq	Two different capture systems- aqueous droplet and micro-bead based 10X Genomics & Microfluidic chip-based Fluigidm C1 Sequencing- Same as for bulk RNA-seq Illumina (HiSeq/ NovaSeq etc),	Most efficient at the level of individual cells (capable of estimating more than 10,000 cells)	High cost, limited number of transcripts can be detected/ identified, no spatial information available	Characterization of multifaceted transcriptional dynamics in heterozygous cell population in complex organ/ tissue network	(28, 29)
**Spatial transcriptiomics**	To explore the global imaging of transcriptional profile of an organ/tissue in situ	**Ex situ Sequencing:** Freshly frozen tissue are cryo-sectioned using OTC and oligo-bead (each having a linker sequence, a spatial barcode, a UMI sequence, and a polyT tail) arrays are placed on the tissue-section slide and slide then transferred to a tube for cDNA synthesis and library preparation and subsequent sequencing. Data are aligned for gene expression matrix and visualized via cluster mapping by barcode location matrix.	10x Visium, Slide-Seq, NanoString GeoMx, HDST etc.	Discover de novo RNA sequence, capable to detect splice-isoforms , SNVs, whole transcriptional data in intact tissue architecture and exact location , 3D visualization possible with multiple sections	Low (10 µm) resolution, cell-cell boundaries not defined, dead spaces between two beads, low detection efficiency particularly for transcripts with low abundance and lack of single cell specific data [however, individual spot (single measurement site) based data can be visualized at single cell resolution by deconvolution tool]	To investigate cellular heterogeneity with spatial information, critical for developmental gene expression histopathology, disease progression, tumour and stem cell micro- environment etc.	(30, 31)
		**Ex situ Sequencing:** Involves direct read out (via in situ cDNA synthesis, rolling amplification and sequencing) the transcript sequence within the tissue. Data are aligned and visualized via reconstitution.					
		**Hybridization based:** Target transcripts are detected by hybridization of complementary fluorescent probes flowed by imaging and decoding the data for visualization.	MERFISH, SeqFISH+ , oligo FISSEQ, Vizgen, 10X Xenium, Nanostring CosMx etc	Higher sub- celluar resolution (400nm) , well defined cell-cell boundaries with proper segmentation, 3D imaging possible without slicing and single RNA molecule is also detectable	Restricted to only known targeted transcripts (on that basis probes are synthesized), Chances of Optical crowding, High cost		

The table is divided into three sections: Bulk RNA-Seq, Single Cell RNA-Seq, and Spatial Transcriptomics.

Despite being critically relevant, comprehensive reviews of recent literature revealing the dynamic landscape of single-cell testicular transcriptomics are currently limited (25, 32). The current study dissects the spermatogenic landscapes viz., SSC/SPC niche, and cellular transcriptional dynamics during the meiotic onset and progression. Finally, this review attempts to reveal the intricate tapestry of testicular development and disorders through next-generation sequencing technologies.

## 2 Methodologies adopted for Sc-RNA-seq

**Figure 2:**
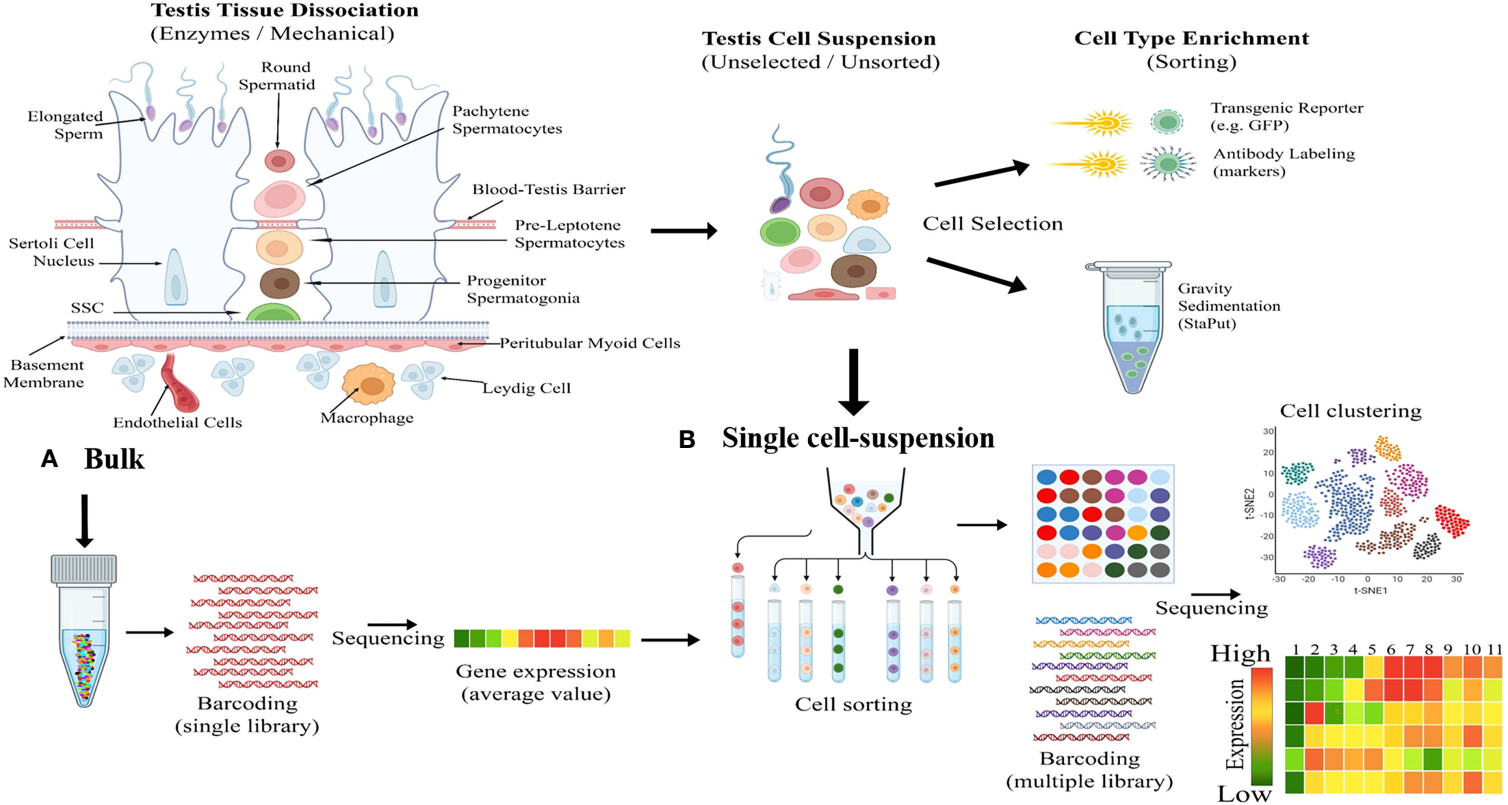
Illustrating fundamental difference between bulk and single-cell RNA sequencing (Sc-RNA-seq). **(A)** Bulk Processing: The left diagram illustrates the enzymatic and mechanical dissociation of testicular tissue into a heterogeneous mixture of cells including Sertoli cells, Leydig cells, spermatogonia, spermatocytes, spermatids etc. Following dissociation, cells are processed in bulk, as shown by the central test tube graphic, including DNA barcoding and sequencing to yield average gene expression profiles. **(B)** Single Cell-Suspension and Enrichment: The top middle flow diagram transitions from an unselected testicular cell suspension to cell type enrichment via methods like transgenic reporters (e.g., GFP) and antibody labeling etc. The bottom middle panel depicts the conversion of the unselected cellular mixture into a single-cell suspension, followed by cell sorting based on type-specific markers, with each cell type represented by a different color. The right side shows the cell clustering outcome, visualized by a t-SNE plot, and the subsequent sequencing of individual cells resulting in a detailed gene expression heatmap, with colors indicating expression levels from high (red) to low (green).

The Sc-RNA-seq generates high-resolution transcriptomic data that precisely identifies minor transcriptomic variations at a single-cell level from a complex organ (33, 34). This tool has significant implications for gonadal development and disorders [viz., infertility, Disorders/Differences of Sex Development (DSDs), and cancer] (26, 27). Sc-RNA-seq produces transcriptomic data employing methods like conventional cellular purification via enzyme-based crude mechanical isolation or transgenic reporter gene/antibody-based sorting of target cells by sophisticated magnetic-activated cell sorter (MACS) or fluorescence-activated cell sorter (FACS) columns (35). **Figure 2** represents the fundamental working principles of bulk and Sc-RNA-seq.

**Figure 3:**
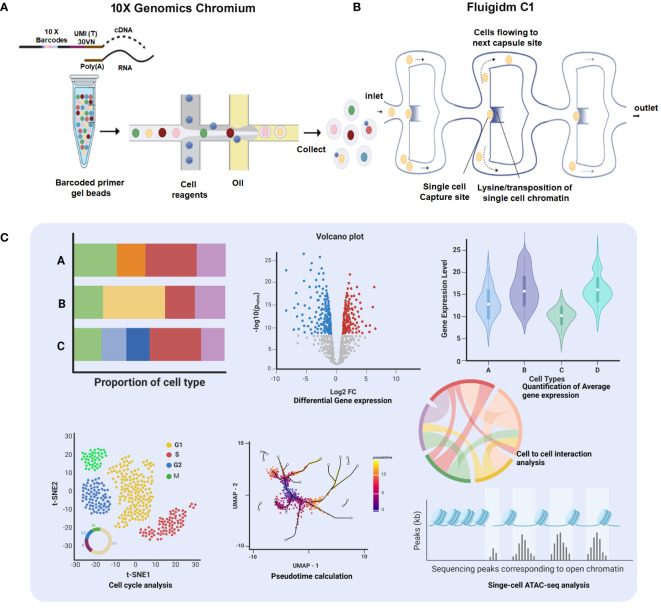
Comprehensive platforms of Single-Cell Transcriptomics. **(A)** 10× Genomics Chromium: The workflow initiates with encapsulation of individual cells with barcoded primer gel beads into oil droplets to generate Gel bead-in-emulsions (GEMs), ready for collecting and processing. Subsequent steps include sequencing, which generates data for proportion of cell type bar charts, a volcano plot showing differential gene expression, and a violin plot depicting quantification of average gene expression across cell types. **(B)** Fluigidm C1: Individual cells are physically captured on microfluidic chips. **(C)** Data Integration and Analysis: The t-SNE plot illustrates cell cycle analysis of individual cells. UMAP with pseudotime calculation provides insights into the developmental trajectory of the cells. The chord diagram highlights cell-to-cell interaction analysis, elucidating the complex interplay between different cell types.

Single-cell isolation and sequencing can be achieved at two different platforms, first droplet-based *10× Genomics Chromium* where individual cells get encapsulated in aqueous droplets along with micro-beads loaded with barcoded primers (36) and second *Fluigidm C1*, where individual cells are physically captured on microfluidic chips (37). After generating Sc-RNA-seq libraries, 10x Genomics utilizes 3′ end-counting unique molecular identifiers (UMIs), a genetic barcode that ensures a distinct cell population is traced and categorized as a specific lineage within a particular tissue/organ (32). In contrast, Fluidigm C1 libraries are automatically generated having full-length transcriptomes with all possible mRNA isoforms/variants (due to alternative splicing/transcription start site, etc.) (37). Notably, a new method *Smart-seq2* has been developed recently having enhanced yield and length of cDNA libraries with better reverse transcription, template switching, and pre-amplification, improving the accuracy of transcript detection at a cheaper cost (38). **Figure 3** schematically illustrates the available Sc-RNA-seq platforms and downstream workflow for data analyses.

**Figure 4:**
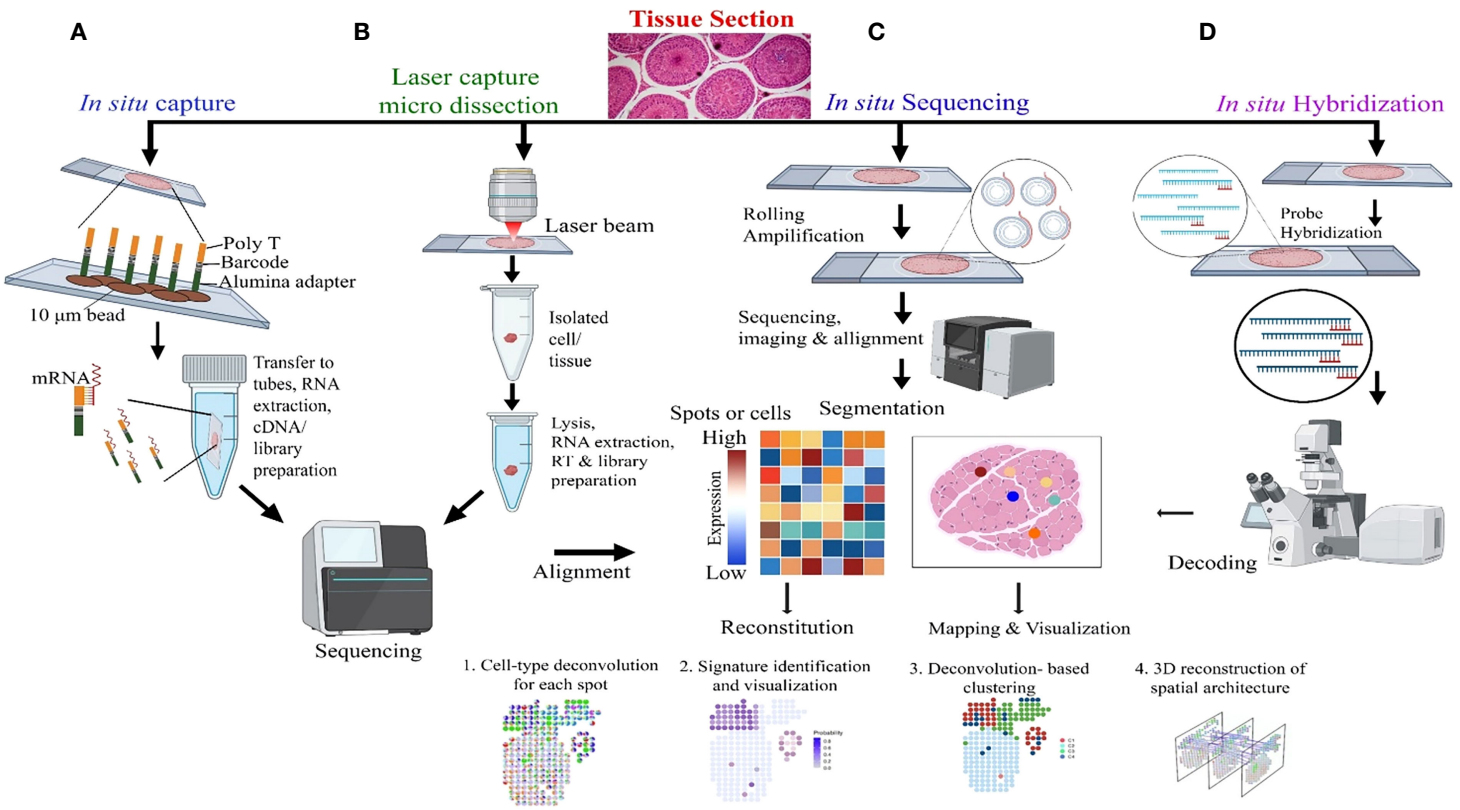
Molecular Profiling of Spatial transcriptomics. **(A)** *In situ* Capture: Cells are captured on a chip with poly-T barcoded beads, allowing for mRNA binding and subsequent steps of RNA extraction, cDNA synthesis, and library preparation, followed by sequencing. **(B)** Laser Capture Micro-dissection: This panel demonstrates the isolation of specific cells or tissue regions using a laser beam, with subsequent lysis, RNA extraction, reverse transcription (RT), and library preparation for sequencing and alignment. **(C)** *In situ* Sequencing: Rolling amplification is performed on tissue sections, followed by sequencing, imaging, and alignment. The heatmap represents gene expression levels in specific spots or cells, which is used for reconstitution, mapping, and visualization of cell types and states. **(D)** *In situ* Hybridization: Hybridization probes target specific RNA in tissue sections, followed by decoding through microscopy, which leads to the identification of mRNA transcripts and allows for 3D reconstruction of spatial architecture within the tissue.

Sc-RNA-seq data are plotted in bi-dimensional space and interpreted by methods like t-Distributed Stochastic Neighbor Embedding (t-SNE) and/or Uniform Manifold Approximation and Projection (UMAP) (33, 34). Although, these methods clearly depict cellular heterogeneity, Sc-RNA-seq data demonstrate cellular condition in a non-functional state as the tissue architecture gets dissociated/disaggregated (39). Therefore, new technology has been developed that captures the spatial transcriptomic data from the tissues via individual-cell laser-capture micro-dissection or multiplexed *in situ* hybridization (39). However, this method also shows limited sensitivity and depends on prior information on types of cells and/or genes to be targeted/analyzed. Recent Slide-seq technology overcomes these limitations and is considered to be the most suitable for generating high-throughput spatial transcriptomic information at 10-μm resolution (40). **Figure 4** depicts the workflow of four popular methodologies being deployed for spatial transcriptomics.

## 3 Fetal testicular morphogenesis

The ontology of testicular germ and somatic cell lineages are developmentally independent (15, 41, 42). The bipotential fetal gonads are formed at the gonadal ridge (GR) during embryonic life [embryonic days (E) 8.0-9.5 in mice and weeks of gestation (W) 3.5-4 in humans] and appear distinctly (by E9.5-10 and W5) with thickening on the ventral side of the mesonephros (43). However, during E6.5- 7.5 and W3 murine and human primordial gem cells (PGCs) emerged from posterior-epiblast and amnion respectively. After getting specified (at E7.5 in mice and W4 in humans), these PGCs start migrating towards the developing GR and finally colonize the nascent gonads by E10.5 in mice and W8 in humans (44). The gonadal PGCs stop proliferation, lose pluripotency to gain competence for meiotic commitment via upregulating RNA binding DAZL protein (45). During E11.5 in mice and W6-7 in humans these nascent gonads undergo sex determination (SD), triggered by *Sry* expression in pre-Sertoli cells of XY gonads (46). The SD event involves step-wise transcriptional cascades generating male (*Sry, Sox9, Fgf9, Amh, Dmrt1* etc.) and female (*Wnt4, Rspo1, Foxl2, Runx1*, etc.) specific robust genetic programming (47). The hallmark of fetal testicular morphogenesis is completed with the formation of the testicular cord and associated vasculature pattern due to selective (XY specific) migration of the mesonephric endothelial cells by E12.5 in mice and W6.5-7 in humans (48, 49). Notably, any developmental defect in the testicular cord formation or abnormality in Sertoli cells (Sc)/Leydig cells (Lc) differentiation directly leads to spermatogenic impairment and infertility during adulthood. Male Gc fail to enter meiosis due to active degradation of Retinoic acid (RA) by CYP26B1 expressed in fetal murine testes from E12.5-13.5 and transformed into mitotically quiescent (G0 arrested) gonocytes (50, 51). During fetal life, E15.5 in mice and W8.5-9 in humans, tubular supportive Sc, PTc, and interstitial fetal Lc (FLc) all are well demarcated (43).

## 4 Exploring cellular heterogeneity in the testis

Testis is a highly complex organ showing remarkable cellular heterogeneity constituted by both germline and somatic cells. Multiple somatic cell types [like nourishing Sc within seminiferous tubules (ST), supportive PTc covering the ST, interstitial (in inter-tubular space) steroidogenic Lc, interstitial macrophages, etc.] and diverse arrays of developing Gc [including pre-meiotic SSCs/SPCs (2n), different stages of meiotic Gc like primary spermatocytes (2n), secondary spermatocytes (n), round and elongated spermatids (n) and post-meiotic spermatozoa (n) etc] within ST contribute the tissue–organization of testis (**Figure 1**). Notably, almost all possible cellular events [like mitosis, meiosis, cellular differentiation, apoptosis, epigenetic remodeling, histone–protamine exchange and meiotic sex chromosome inactivation (MSCI) etc.] are evident in testis (52). **Table 2** summarizes distinct/key cell markers of individual testicular cells with respect to the appropriate functional significance regulating male fertility. We here have discussed individual major cell–types contributing to the tissue organization of adult testis.

**Table 2:** Essential Genes Influencing Spermatogenesis Identified Across Multiple Single-Cell RNA Sequencing Research Studies.

S. No.	Specific Marker/ Enriched Transcript	Full Name	Specific Cell type	Key Function/ Role
**1.**	**ID4**	Inhibitor of Differentiation 4	SSCs / SPCs and Undifferentiated Spermatogonia (Spg)	Typical SSC marker
**2.**	**GFRA1**	GDNF family receptor α-1	SSCs/ SPCs and Undifferentiated Spg	Critical for SSC self-renewal and differentiation.
**3.**	**MAGEA4**	Melanoma Antigen Gene Family A, Member 4	Undifferentiated and differentiated Spg	Essential for normal testicular development and function, with its expression pattern and function tightly regulated to ensure proper spermatogenesis and male fertility.
**4.**	**SYCP3**	Synaptonemal Complex Protein 3	First meiotic prophase, including preleptotene, leptotene, and zygotene spermatocytes (Sct)	Assembly and stability of the synaptonemal complex, as well as for the progression of meiotic recombination and synapsis between homologous chromosomes.
**5.**	**DMC1**	DNA Meiotic Recombination Protein 1	First meiotic prophase	Key component of the meiotic recombination machinery and is specifically involved in the repair of DNA double-strand breaks (DSBs) that occur during meiosis
**6.**	**PIWIL1**	Piwi Like RNA-Mediated Gene Silencing 1	Leptotene, pachytene,and zygotene Sct	*Maintenance of genomic integrity through transposon silencing*
**7.**	**PGK2**	phosphoglycerate kinase	Spermatids	Critical for meiotic germ cell metabolism
**8.**	**ACR**	Acrosin	Pachytene Sct to postmeiotic spermatid stage	Also known as the *Acrosin* gene, plays a crucial role in testicular development by encoding the Acrosin protein
**9.**	**GAPDHS**	Glyceraldehyde-3-Phosphate Dehydrogenase, Testis-Specific	Primary spermatocytes	Essential for regulating energy metabolism and sperm maturation processes
**10.**	**PRM1**	Protamine 1	Spermatid	Ensures the proper packaging and condensation of DNA within sperm cells, which is essential for male fertility and successful reproduction.
**11.**	**SRY**	Sex-Determining Region Y	Sertoli cells	Master regulator of male sex determination
**12.**	**SOX9**	SRY (Sex Determining Region Y)-Box 9	Sertoli cells	Critical role in promoting testicular morphogenesis Sertoli cell differentiation
**13.**	**WT1**	Wilms Tumor 1	Sertoli cells, germ cells, and interstitial cells (Leydig cells)	Differentiation and maintenance of Sertoli cells.
**14.**	**DMRT1**	Doublesex and Mab-3 Related Transcription Factor 1	Sertoli cells, Germ cells	Maintains testicular differentiation by suppressing Foxl2
**15.**	**FGF9**	Fibroblast Growth Factor 9	Sertoli cells	Critical role in promoting testicular morphogenesis Sertoli cell differentiation
**16.**	**ALDH1A1**	Retinaldehyde dehydrogenase 1A1	Leydig cells	Catalyzes the conversion of retinaldehyde to retinoic acid, a form of vitamin A. Retinoic acid serves as a signaling molecule that regulates the expression of genes involved in germ cell differentiation and meiosis initiation. Additionally, ALDH1A1 has been implicated in protecting germ cells from oxidative stress, thereby promoting their survival and proper development within the testes.
**17.**	**AMH**	Anti-Müllerian Hormone	Sertoli cells	Regression of the Müllerian ducts, preventing the development of female reproductive structures.
**18.**	**IGFBP5**	Insulin-Like Growth Factor Binding Protein 5	Sertoli and Leydig cells	Bioavailability and activity of insulin-like growth factors (IGFs), which are involved in testicular development and spermatogenesis
**19.**	**INSL3**	Insulin-Like 3	Sertoli cells	Descent of the testes during fetal development
**20.**	**RSPO1**	R-Spondin 1	Leydig cells	Regulation of Wnt signaling pathways, which play important roles ovarian differentiation
**21.**	**KITL (KIT)**	Kit Ligand (Stem Cell Factor)	Sertoli and Leydig cells	Induction of proliferation/ differentiation / survival of SSC ‘ Spermatogonial cells
**22.**	**NR5A1 (SF1)**	Nuclear Receptor Subfamily 5 Group A Member 1 (Steroidogenic Factor 1)	Sertoli and Leydig cells	Upstream regulator of *Sry*, induces steroidogenesis, sexual differentiation.
**23.**	**BMPR2**	Bone Morphogenetic Protein Receptor Type 2	Sertoli cells, Leydig cells, and germ cells	Regulation of cell growth, differentiation, and apoptosis.
**24.**	**AR**	Androgen Receptor	Sertoli cells, Leydig cells, and germ cells	Binds with testosterone, critical for testicular development, spermatogenesis, and male sexual differentiation.
**25.**	**CYP17A1**	Cytochrome P450 Family 17 Subfamily A Member 1	Leydig cells	It is involved in the biosynthesis of androgens, such as testosterone and dehydroepiandrosterone (DHEA).
**26.**	**HSD3B1**	Hydroxy-Delta-5-Steroid Dehydrogenase, 3 Beta- and Steroid Delta-Isomerase 1	Leydig cells	Catalyzes the conversion of pregnenolone and 17-hydroxypregnenolone to dehydroepiandrosterone (DHEA) and androstenedione, respectively, which are precursors for testosterone biosynthesis.
**27.**	**LHCGR**	Luteinizing Hormone/Chorionic Gonadotropin Receptor	Leydig cells and germ cells	Mediates the effects of luteinizing hormone (LH) on Leydig cells, stimulating testosterone production.
**28.**	**ACVR2A**	Activin A Receptor Type 2A	Sertoli cells, Leydig cells, and germ cells	Mediates the effects of activin and other ligands on cell growth, differentiation, and apoptosis.
**29.**	**DHH**	Desert Hedgehog Homolog	Sertoli cells	Critical role in testicular development, Sertoli cell differentiation, germ cell proliferation and spermatogenesis.
**30.**	**EFNB2**	Ephrin B2	Sertoli cells	Involved in cell-cell signaling and adhesion processes during testicular development and spermatogenesis.
**31.**	**NOTCH4**	Notch Receptor 4	Sertoli cells, Leydig cells, and germ cells	Critical in Leydig cell fate determination, differentiation, and proliferation.
**32.**	**PDGFB**	Platelet-Derived Growth Factor Subunit B	Leydig cells	Involved in cell growth, differentiation, and angiogenesis.
**33.**	**CXCR4**	C-X-C Motif Chemokine Receptor 4	Sertoli cells, Leydig cells, and germ cells	Homing of gonocytes/ SSC to basement membrane to establish SSC pool/ niche
**34.**	**GDNF**	Glial Cell Line-Derived Neurotrophic Factor	Sertoli cells, and peritubular cells	Regulation of SSC self-renewal and differentiation.
**35.**	**GJA1**	Gap Junction Protein Alpha 1	Sertoli cells and germ cells	Forms gap junctions between adjacent Sertoli cells to form BTB.
**36.**	**FGFR1/R2/R3**	Fibroblast Growth Factor Receptor 1/2/3	Sertoli cells, Leydig cells, and germ cells	Mediates the effects of fibroblast growth factors (FGFs) on cell growth, differentiation, and survival.
**37.**	**BMP4/8b**	Bone Morphogenetic Protein 4/8b	Sertoli cells	Critical for testicular development, PGC specification, germ cell differentiation, and spermatogenesis.
**38.**	**HMGA1**	High Mobility Group AT-Hook 1	Sertoli cells, Leydig cells, and germ cells	Involved in chromatin remodeling, gene regulation, and cell differentiation.
**39.**	**SMAD1/5/7**	SMAD Family Member 1/5/7	Sertoli cells, Leydig cells, and germ cells	Regulation of transforming growth factor-beta (TGF-β) signaling pathways, which play important roles in testicular development, germ cell differentiation, and spermatogenesis.
**40.**	**STRA8**	Stimulated by Retinoic Acid Gene 8	Meiotic germ cells	Meiotic gatekeeper
**41.**	**DMC1**	DNA Meiotic Recombination Protein 1	Meiotic germ cells	Involved in the repair of DNA double-strand breaks during meiotic recombination, ensuring proper chromosome segregation and genetic diversity in sperm cells.
**42.**	**RAD51**	RAD51 Recombinase	Germ cells	Involved in homologous recombination repair of DNA double-strand breaks during meiosis and DNA repair processes.
**43.**	**ADAMTS2**	ADAM Metallopeptidase with Thrombospondin Type 1 Motif 2	Sertoli cells, Leydig cells, and germ cells	Involved in extracellular matrix remodeling and tissue homeostasis.
**44.**	**CSF1**	Colony Stimulating Factor 1	Sertoli cells	Regulation of SSC and macrophage differentiation and function
**45.**	**CDKN2D**	Cyclin-Dependent Kinase Inhibitor 2D	Sertoli cells, Leydig cells, and germ cells	Involved in the regulation of cell cycle progression and proliferation
**46.**	**DAZL**	Deleted in Azoospermia Like	germ cells	Germ cell licensing or commitment towards meiosis
**47.**	**RHOX5**	Reproductive Homeobox 5	Sertoli cells and germ cells	Critical Androgen responsive gene supports male fertility.
**48.**	**RHOX 10**	Reproductive Homeobox 10	SSCs / SPCs and Undifferentiated Spg	Maker of SSC , important for self-renewal
**49.**	**SLC25A4**	Solute Carrier Family 25 Member 4	Sertoli cells, Leydig cells, and germ cells	Involved in the transport of metabolites across mitochondrial membranes and energy metabolism.
**50.**	**FSHR**	Follicle-Stimulating Hormone Receptor	Sertoli cells	Promote Proliferation of Sertoli cells and support pre-meiotic germ cell differentiation.

### 4.1 The germ-line cell types

The Gc residing within ST passes through a series of developmental stages [spermatogonia (2n), primary and secondary spermatocytes (n), round and elongated spermatids (n) etc] culminating in the production of mature sperm. Here we discuss different stages of developing Gc.

#### 4.1.1 Primordial germ cells

*Primordial germ cells (PGCs)* represent the earliest identifiable precursors of Gc. PGCs originate via direct induction of amnion and/or posterior-epiblast (PE) cells from adjacent extra-embryonic ectoderm (EEE) and visceral endoderm (VE) by morphogens like bone morphogenetic proteins (*Bmp4* and *Bmp8b*), Activin A and transcription factors like *Blimp1* (Transcriptional repressor B lymphocyte induced maturation protein 1, also known as PR domain-containing protein 1, *Prdm1*), *Prdm14* (PR domain zinc-finger protein 14) and *AP2γ* (activating enhancer-binding protein 2γ or *Tfap2c*) and *Sox17* (only in humans) etc. (44). The PGCs migrate to colonize the developing GR and subsequently gain the meiotic commitment (lose pluripotency by upregulating DAZL) by E12.5 in mice and W8-9 in humans (50).

#### 4.1.2 SSCs

*SSCs* are established from gonocytes post-birth (postnatal age 2-3 days in mice) which guarantee the continuity of sperm production throughout adult life (53). In rodents and primates, the duration of spermatogenesis varies among species, (in days; man 64, rhesus monkey 42, mouse 35, and rat 52) typically spanning 35 to 70 days (10, 54). In rodents A_s_ (A single spermatogonia) and in primates A_pale_ (proliferative stem/progenitor pool) or A_dark_ (slow diving reserve stem pool) are considered to be the functional SSC populations respectively (13, 14). The key balance between self-renewal and differentiation of these cells remains critical for spermatogenic maintenance throughout adult life (13). The Sc and PTc contribute to establishing the micro-environment of SSC that governs such regulated balance (55).

#### 4.1.3 SPCs

*SPCs* are constituted with undifferentiated Spermatogonia (Spg), type A_paired_ or A_aligned_ connected with common cytoplasmic bridges. In rodents, these pre-meiotic (mitotic) Gc exhibit clonal fragmentation and get differentiated into a transit amplifying (TA) population (A1 to A4) induced by RA and then further transformed to differentiated Spg type B stage having meiotic fate (56). In primates, no such TA population is observed however, in rhesus monkeys (not human) 4 categories of Spg B (B1,B2,B3,B4) are found. In rodents, RA further drives the meiotic entry of Spg B by inducing *Stra8*, *Rec8* and *Meiosin* expressions in these cells (50, 57).

#### 4.1.4 Meiotic Gc

*Meiotic Gc* represent the most critical stage of spermatogenesis (58) with two successive divisions. In the first division, diploid (2n) primary spermatocytes transform into haploid (n) secondary spermatocytes followed by in the second division, these spermatocytes further divide to produce round spermatids. Meiotic Gc are pivotal in reducing the chromosome number by half, ensuring that the resulting sperm cells possess the correct genetic material for fertilization upon union with an egg cell. The juxtacrine testosterone (T) signaling through Sc regulates the meiotic progression (59).

#### 4.1.5 Post-meiotic Gc

**Figure 5:**
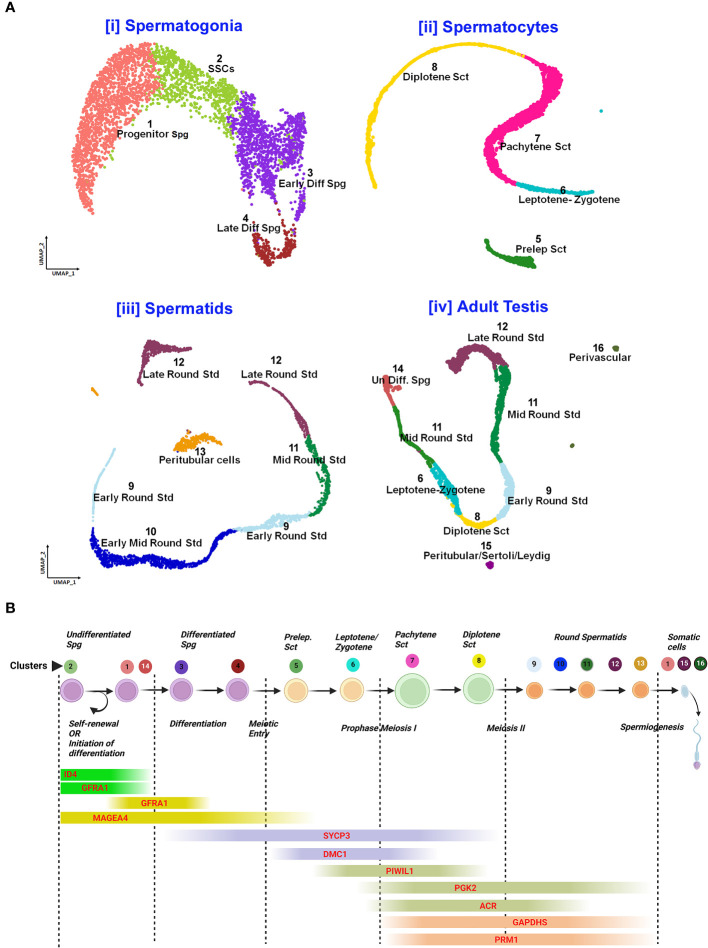
Single-cell analysis of spermatogenesis reveals developmental trajectories and gene expression profiles. **(A)** UMAP visualization of cell clusters from testicular single-cell RNA sequencing: Cell Differentiation Trajectories: [i] Spermatogonia clusters 1-4 (Progenitor to Late Differentiated), [ii]. Spermatocytes stages 5-8 (Preleptotene to Diplotene), [iii]. Spermatids 9-12 (Early to Late Round), [iv]. Adult Testis Cells 13-16 (Undifferentiated to Perivascular). **(B)** Gene Expression Markers throughout spermatogenic progression: Sequential expression from self-renewal (GFRAL) to spermiogenesis (PRM1).

**Figure 6:**
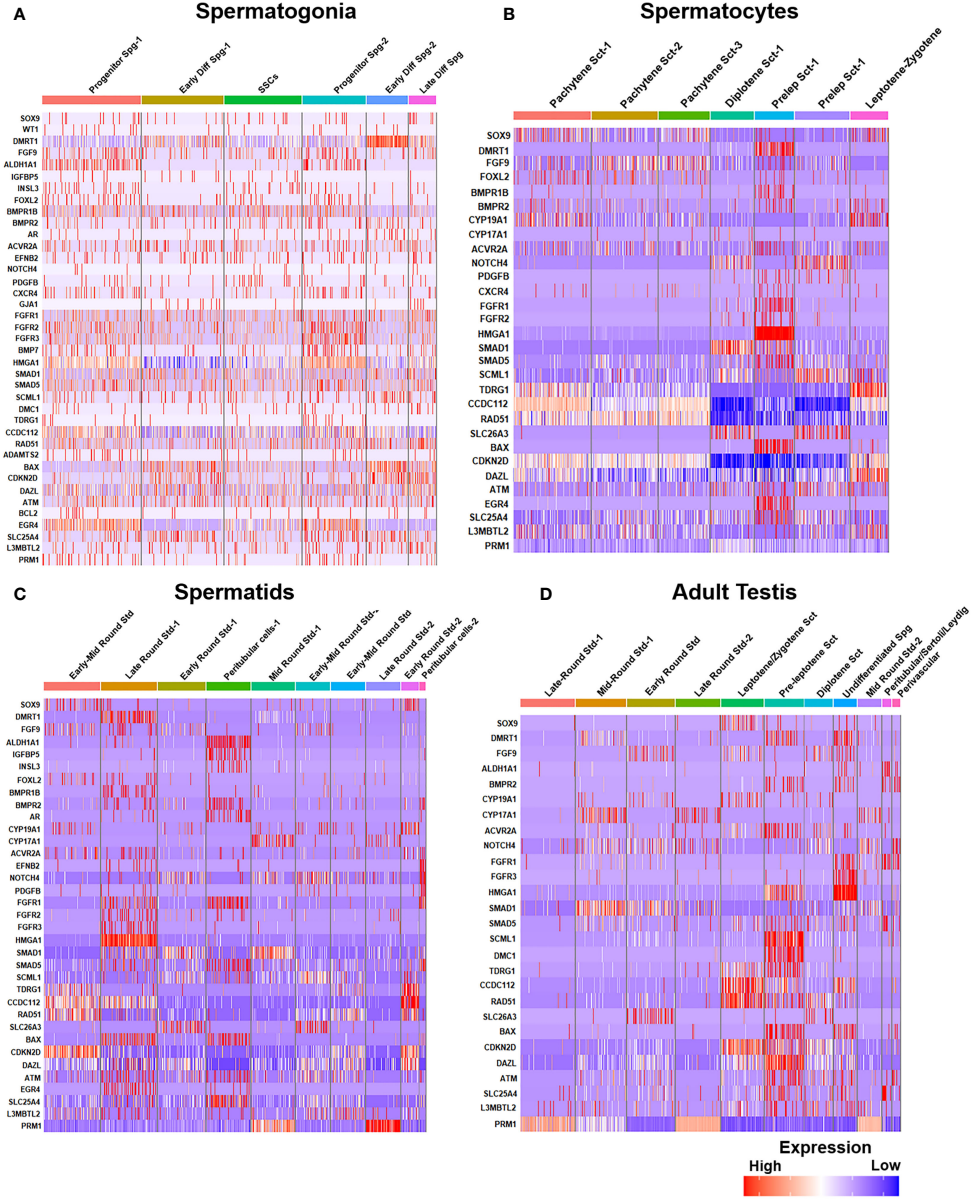
Gene expression profiles during human spermatogenesis. **(A)** Gene expression heatmap of spermatogonia: The heatmap illustrates the gene expression levels at different steps of spermatogonial development, from pre-meiotic to SSCs. **(B)** Gene expression heatmap of spermatocytes: Displays the gene expression patterns during the meiotic stages (spermatocytes, from pre-leptotene to post-meiotic). **(C)** Gene expression heatmap of spermatids: Depicts variations of gene expression in spermatids, highlighting the transition from early to late spermatid stages. **(D)** Comparative expression heatmap of adult human testis: Compares gene expression across different cell types and stages of sperm development, including leptotene and zygotene spermatocytes and diplotene to secondary spermatocytes.

*Post-meiotic Gc* maturation mainly involves the transformation of a round spermatid to an elongated structure and such elongated spermatids get condensed and further mature into spermatozoa. In this step, the histone–protamine transition is observed leading to massive hetero-chromatinization leading to transcriptional silencing. However, the functional maturation of male gamete occurs with spermiogenesis, which involves complex cellular changes, viz., development of acrosome and flagellum, etc. Unraveling this metamorphosis is crucial for addressing male fertility concerns and developing strategies for male reproductive health and contraception (60). **Figure 5** elucidates the testicular transcriptional dynamics concerning complex cellular heterogeneity. **Figure 5A** exhibits the differential degrees of heterogeneous/diverse gene expression profiles observed in multiple developmental stages of human (i) spermatogonia, (ii) spermatocytes, (iii) spermatids, and (iv) the entire adult human testis. **Figure 5B** is the graphical representation of developing Gc-specific marker expression during the entire spermatogenic progression. **Figure 6** illustrates the comparative heat maps showing the expression profiles of critical genes involved/associated with different developmental/maturational stages of (A) spermatogonia (B) spermatocytes (C) spermatids and (D) whole adult testis.

Notably, *Dazl* (essential for gametogenic competence to Gc) expression was found to be consistent in all stages of spermatogonial development [like SSCs, progenitor spermatogonia (Spg)-1,2, early differentiating Spg-1,2 and late differentiating Spg] (**Figure 6A**). However, *Dazl* expression gets upregulated in high leptotene-zygotene spermatocytes (Sc1-1); declined in subsequent pachytene stage. Essential meiotic genes like *Dmc1, Scml1* and *Rad51* were found to be elevated in pre-leptotene stage, whereas, RA synthesizing genes like *Aldh1a1* was highly enriched in somatic Sc/PTc. Finally, *Dmrt1*, a critical regulator testicular development was found to get augmented in early differentiating Spg-2, pre-leptotene Sc1-1 and late round spermatid (Std1) (**Figure 6**).

### 4.2 The somatic cells

Five distinct somatic cell types (that undergo mitosis but not meiosis) provide the critical structural and nutritional support are discussed in the following section.

#### 4.2.1 Sertoli cells

*Sertoli cells (Sc)* are the major somatic cells that are the target of both FSH and T, known to dictate spermatogenesis by providing direct structural and nutritional support to all stages of developing Gc within the ST. Sc nurture and regulate the meiotic progression and completion of Gc. Sc-Sc tight junctions establish the blood-testis barrier (BTB) essential for Gc development and their directional movement towards the tubular lumen (61). FSH is critical for Sc proliferation and expansion of the pre-meiotic Gc population, whereas T governs the BTB formation, dynamics and Gc meiosis (4, 62). Sc derived glial cell line-derived neurotrophic factor (GDNF) and stem cell factor (SCF) play crucial role in determining the SSC fate decision from self-renewal to differentiation. Fetal Sc originate from the coelomic epithelium (63) and get specified (at E11.5 in mice and W6-7 in humans) by *Sry* driven *Sox9* expression (43).The development of Sc is distinctly divided into three phases, immature (during fetal/neonatal/perinatal phase), maturing (during juvenile/pre-pubertal life) and mature (at puberty and adulthood) (64–66). Hormonal (FSH & T) stimulations to fetal/neonatal/infant Sc, show limited responses like self-proliferation and local expansion of pre-meiotic Gc but are found to be insufficient to induce robust onset of spermatogonial differentiation. The functional immaturity in terms of hormonal responsiveness of younger Sc is considered the underlying cause. Restricted plasma membrane localization of FSH-Receptor (*Fsh-r*), inadequate expression of associated Gαs sub-units and poor binding of T with Androgen Receptor (*Ar*) have been demonstrated in immature Sc during infancy (67–70). Therefore, the functional maturation of pubertal Sc is found to be developmentally critical for inducing Gc differentiation. Such pubertal maturational event in Sc become a prerequisite for spermatogenic onset and male fertility (15, 65). This is further evident by the presence of persistent immature Sc in adult testes that results in severe oligozoospermic conditions with infertility in rodents (71–75) and in humans (64, 76–79).

#### 4.2.2 Peritubular cells

*Peritubular cells (PTc)* constitute the outermost covering of the ST and further provides structural support by creating peristaltic waves through their contractile elements, potentially prompting fluid movement within the tubule lumen to facilitate the expulsion of spermatozoa (80). PTc are the targets of T signaling and shown to promote Sc maturation. Furthermore, crosstalk between PTc with adjacent Lc play a vital role in spermatogenic development. Along with Sc, these cells govern the maintenance of the SSC micro-environment (81, 82).

#### 4.2.3 Leydig cells

*Leydig cells (Lc)* are the key steroidogenic cells located within the inter-tubular interstitium and the sole target of LH for T biosynthesis. Lc are critical for male reproductive development viz., differentiation of the genital tract, fetal virilization, pubertal maturation and regulation of fertility during adulthood (82, 83). Two [fetal (FLc) and adult (ALc)] and four (fetal, neonatal, pubertal, and adult) distinct populations of Lc are reported in mice and primates with differential morphology and functions respectively (41). In mice, FLc exhibit dual origins (both coelomic epithelium and notch-active Nestin-positive perivascular cells located at the gonad–mesonephros borders) and first detected as *Nr5a1* (also known as Ad4BP/SF-1) positive cells on E12.5 (W9-10 in humans). Murine FLc lack HSD17β3 enzyme and thereby produce only androstenedione (precursor of T) and promote the initial virilization and differentiation of male genitalia. Post-birth by P (post-natal age in days) 7-12, FLc undergo periodic regression and replaced by T-producing ALc during P18-25. Nestin-positive perivascular cells and FLc are the progenitor populations for ALc (84). Recent research indicates that 5-20% of FLc are maintained in adult testis (37). T acts on ALc, PTc and Sc to promote spermatogenesis, however, Gc do not express *Ar*. Sc shown to be the most critical target of T signaling for regulating male fertility (85). Although the extent of LH responsiveness of ALc remain unaltered with testicular aging, overall T production declines with testicular aging and testicular metabolic complications (86–88).

#### 4.2.4 Macrophages

*Macrophages*, (being CSF1R^+^, CD206^+^ MHCII^-^) are present in the testicular interstitium and regulate the steroidogenic activity of ALc (84, 89). Another distinct population of adult macrophages (CSF1R^-^, CD206^-^, MHCII^+^) has been observed at various patches of the peritubular region which actively contributes to the SSC niche and promotes spermatogonial differentiation (80, 90). Intriguingly in fetal life, yolk-sac-derived murine macrophages (at E7.5) play a critical role in directing the selective migration of mesonephric endothelial cells towards developing XY gonads (not XX) and directs testicular cord (at E12.5) formation (49). Recent findings indicate that monocytes derived from hematopoietic stem cells (HSCs) of fetal liver get colonized in embryonic mouse testes in Sc dependent manner and further differentiate into testis resident macrophages during postnatal life (84). During adulthood, CD206^+^ interstitial macrophages promote ALc proliferation and steroidogenesis and thereby indirectly regulate male fertility. Furthermore, these macrophages also maintain the immunosuppressive environment within the testis (91).

#### 4.2.5 Endothelial cells

*Endothelial cells (ECs)* form the border walls of blood vessels, facilitating nutrient exchange for developing Gc/sperm. Dysfunction in these cells can affect sperm development and overall testicular health, impacting fertility. During murine testicular morphogenesis, a key feature involves sex-specific vascularization where endothelial cells migrate from the neighboring mesonephros into the fetal XY gonads (during E11-12.5), encircling Sc-Gc clusters and prompting the formation of seminiferous cords. These cells are potent sources of vascular endothelial growth factor A (*Vegf A*) critical for tissue remodeling/homeostasis (92). ECs also contribute to the SSC niche by producing GDNF that sustains SSCs during extended culture periods (93).

**Figure 7:**
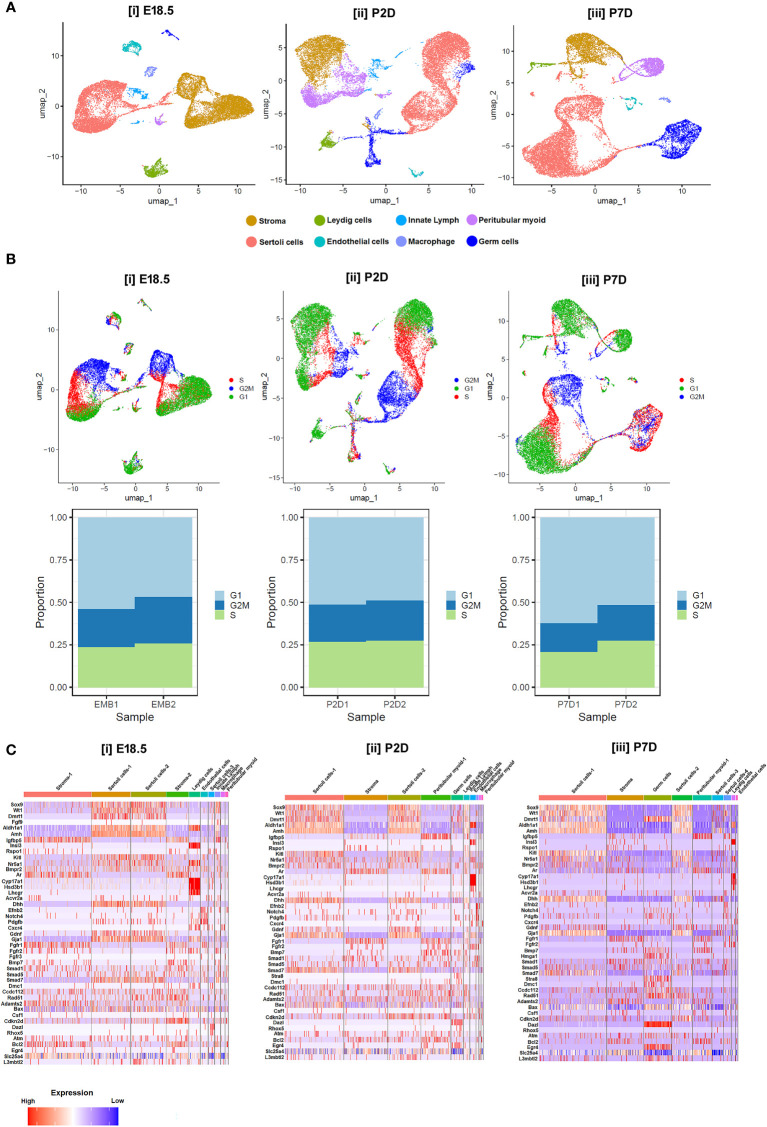
Single-cell transcriptomic analysis of testicular cells at different developmental age groups. **(A)** UMAP visualizations representing the cellular composition of testicular cells at (i) embryonic days 18.5 (E18.5), (ii) postnatal days 2 (P2D), and (iii) postnatal days 7 (P7D). Each dot represents a single cell, color-coded by identified cell types: stroma (cyan), Leydig cells (yellow), Sertoli cells (dark blue), endothelial cells (green), innate lymphoid cells (purple), peritubular myoid cells (red), macrophages (light blue), and germ cells (orange). **(B)** UMAP plots displaying cell cycle phases for testicular cells on E18.5, P2D, and P7D. Cells are color-coded according to their phase in the cell cycle: G1 (blue), S (red), G2/M (green), and cells not in cycle (black). Arrows indicate the direction of the developmental trajectory. **(C)** Heatmaps illustrating the expression profiles of selected marker genes across different cell types on E18.5, P2D, and P7D. Gene expression levels are represented by a gradient from low (blue) to high (red) expression. Beneath each UMAP plot in B, stacked bar charts show the proportion of cells in each cell cycle phase for two replicates at each developmental stage (EMB1, EMB2, P2D1, P2D2, P7D1, P7D2).

Notably, moderate expressions of *Dmrt1, Sox9, Wt1, Amh, Dhh* and *Aldh1a1* were found in murine Sc from E18.5 to P2D upto P7D. However, the transcript levels of the genes essential for induction of Gc differentiation like *Scf/Kit-ligand, Gdnf* and *Csf-1* etc, remained low during this time of testicular development. In consistent with previous reports, Lc specific *Insl3, Cyp17a1, Hsd3b1, Lh/cg-receptor* and PTc restricted *Ar* expressions were observed from E18.5 to P7D; whereas meiotic gatekeeper *Stra8* expression was enriched at Gc on P7D (**Figure 7C**). **Figures 7A, B** show age-dependent dynamic gene expression profiles observed in different cell types during three [(i) E18.5, (ii) P2D (post-natal 2 days of age) & (iii) P7D (post-natal 7 days of age)] distinct developmental phases of murine testicular maturation. **Figure 7C** depicts the comparative heat maps revealing the expression profiles of key spermatogenic genes in different cell types during (i) fetal (E18.5) testicular developmental and neonatal [(ii) P2D, (iii) P7D] testis maturation.

## 5 A comprehensive review of literature on testicular Sc-cell-RNA-seq revealed the molecular basis of testicular dysfunctions/disorders

The literature search was conducted during December – 2023- February 2024. Initially, systematic inquiries were performed in the PubMed database. Given the review’s primary focus on “single-cell RNA sequencing” and “Testis”, a tailored search strategy was applied to connect these two aspects. In detail, concerning the former aspect, the terms “single-cell” (term A) or “Sc-RNA-seq” (term B) were designated. Each of these terms was paired with one of the subsequent testis-related (the latter aspect-related) or animal model-related search terms, which included “testis” (term 1), “spermatogenesis” (term 2), “testicular development” (term 3), “azoospermia” (term 4), “Klinefelter Syndrome” (term 5), “cryptozoospermia” (term 6), “oligozoospermia” (term 7), “asthenospermia” (term 8), “teratospermia” (term 9), “orchitis” (term 10), “cryptorchidism” (term 11), “male and gonad” (term 12), “Humans” (term 13), “Monkey” (term 14), “Primates” (term 15), “mouse” (term 16), “Rat” (term 17), “Testicular diseases” (term 18), and “Testicular atlas” (term 19).

**Figure 8:**
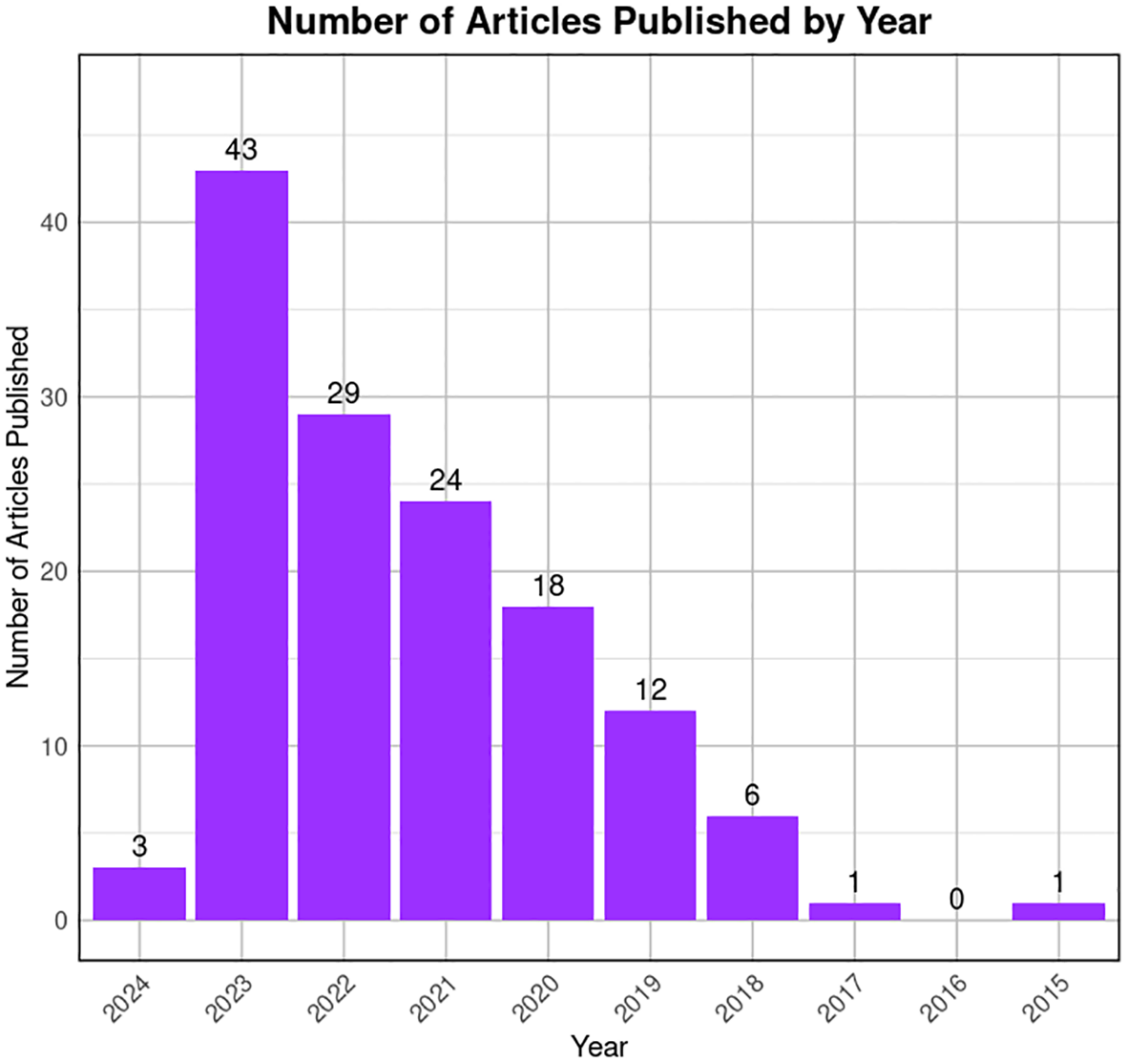
Trends in publication on testicular Sc-RNA-seq studies. The bar graph illustrates the number of articles published by year, from 2015 to 2024 available at PubMed. A noticeable peak is observed in 2022, followed by a decline in subsequent years. The x-axis represents the years, while the y-axis quantifies the number of articles published. The data suggests a period of heightened research activity in 2022, with a gradual decrease in the volume of publications thereafter.

To reduce the likelihood of overlooking pertinent studies, particularly preprint studies, an additional search is conducted on the GEO dataset (https://www.https://www.ncbi.nlm.nih.gov/geo/) employing combinations of refined above search terms. The searches yielded approximately ~132 records from the last 10 years (2015–2024). Among these, 60 articles contained original Sc-RNA-seq data (from at least one donor) related to human testicular samples, while the remaining 68 articles involved re-analyzed data from prior publications or the datasets from other primates, rodents, livestock animals, or non-mammalian species. Starting in 2015, Guo et al. pioneered Sc-RNA-seq on 233 individual male and female human PGCs from 15 embryos between 4 and 19 weeks of gestation (94), followed by two studies in 2017 that sequenced adult testicular cells (95, 96). Interestingly, despite being from different teams, both adult studies focused on spermatogonia. Since 2018, numerous studies have conducted Sc-RNA-seq on human testicular samples, encompassing both prenatal and postnatal stages, normal and abnormal testes. **Figure 8** represents the timeline of literature published/available in PubMed. **Table 3** is the chronological summary of original research articles published on Sc-RNA-seq data for normal testicular samples collected from (A) primates (human and monkeys) and (B) rodents. **Table 4** discusses country-wise Sc-RNA-seq data on clinical human samples with multiple forms of impaired spermatogenesis/infertility reported so far. Notably, **Tables 3**, **4** will serve as a critical repositories/valuable resource for Sc-RNA-seq datasets specific to distinct testicular cell types generated to date.

Table 3Summarizing the key findings of mammalian testicular single-cell RNA-seq datasets. (A). Normal healthy Human/ Monkeys. (B). Exclusively Rodents.

**A:** Normal healthy Human/ Monkeys

S. No	Year	Species	Num ber of Donar(s)	Sample Target Group (specific cell types)	Number of Cells examined	Age of tissue	GEO Accession Number	Tool and Sequencing Platform	Major Findings/ Critical Outcome	References
1	2015	Human	15 embryos from 9 donars	Primordial Germ Cells (PGCs)	197-319 cells	Fetal gonads at 4 to 19 weeks of gestation		Tang method followed by Illumina HiSeq2000/25 00 sequencer for 100 bp or 150 bp paired- end sequencing.	Inactivated X chromosomes get reactivated in PGCs by 4 weeks of age, gene expression pattern found to be homogeneous during 4 and 11 weeks of embryonic age in PGCs and global erasure of DNA methylation gets completed by 10 to 11 weeks.	(94)
2	2017	Human	5	SSEA4^+^ SSC and differentiating c- KIT^+^ spermatog onia	Single cell preparation 15,000- 20,000 out of which 175(92 filtered) analysed	Adult Unknow n	GSE92280	Fluidigm C1 followed by Illumina HiSeq2500	Sc-RNA-seq clustering analysis reveals four cellular/developmental states during SSC differentiation, e.g.- *Cluster A* for *state 1* key markers Id4, Etv5 (major transcription factors) and Txnip (for inhibiting glucose update); *Cluster B* for *state 2 having Fgfr1/2 , Bmpr etc; Cluster C* for *state 3* with Ndufa/b (metabolic mitochondria), Sohlh2, Nr5a1, Thgbr1 (differentiation) , lsm3/4/6 (RNA splicing) *Cluster D* for *state 4* with Cdc45, Rec8, Fanca (cell cycle, DNA repair) etc	(95)
3	2017	Human	Spermatogonial arrest (n = 1) and normal spermatogenesis (n = 7)	Spermatogonia	20 and 105	Unknown	GSE91063	Tang method/Shallow RNA-seq followed by Ion Torrent PGM	Prominent heterogeneous transcriptomic profiles of *Oct4, Utf1, Magea4, Boll, Prm2, Ddx5, Tspy1, Eef1a1* and *Ngn3* in spermatogonial cells either with spermatogenic arrest (20 cells) or in normal spermatogenesis (105 cells).	(96)
4	2017	Human	29 (17 female and 12 male) embryos	Human fetal germ cells (FGCs)	2,167 single FGCs for analysis 1204 (1068 filtered)	21 weeks of gestation	GSE86146	Modified smart-seq2 followed by Illumina HiSeq 2500	Reciprocal BMP and Notch signaling network found between FGCs and supportive gonadal cells, male FGCs develop through stages of migration, mitosis, and cell-cycle arrest (without RA signaling and meiosis)	(97)
5	2017	Human	6 (all are biological fathers)	Germ cell subtypes	500	38-52 years	SRP069329	Laser capture microdissection, 5-µm-thickness of fixed testis; Illumina HiSeq 2000	This study has identified over 4000 genes including the expression of 110 RNA-binding proteins and 137 long non-coding RNAs, previously unknown in spermatogenesis. Minimal transcriptional changes found between quiescent Adark to active Apale spermatogonia.	(98)
6	2018	Human	4 adults and 2 infants	Human infant and adult testis	~650,000 out of which 7830 filtered	17-25 years in young adults, 13 months for infants	GSE120508	10× Genomics Chromium Controller followed by Illumina HiSeq 2500	This study reveals **>** 8000 differentially regulated genes and uniquely explored multiple transposable elements (TE) like LTR12C/D/E, SVA_D, AluYa5, LTR10A, and LTR40C elements, and long non-coding RNAs during multiple steps of spermatogenesis. Five distinct states of spermatogonia (including a novel early SSC state, termed State 0) with discrete transcriptional status and developmental plasticity has been reported.	(99)
7	2018	Human and mice	1610 (2 adult normal men, 7 OA and 1 NOA)	Spermatogonia, meiotic germ cells and haploid spermatids Spermatogonia, meiotic germ cells and haploid spermatids	4,651 and 7,134 spermatogenic cells from mice and men for analysis 36451 for 10x and 635 for C1	Organ donor 34.3 ±7.2 years) than biopsy patients (42.4 ± 1.2years)	GSE108970, GSE108974, GSE108977, GSE109049, GSE109033, GSE109037	Both 10x Genomics and Fluidigm C1 followed by Illumina NextSeq 500; Illumina HiSeq 3000	This study reports Sc-RNA-seq of >62,000 spermatogenic cells from mice and humans showing phylogenetic resemblance in SSC fate regulation. The hepatic stellate cell activation pathway is shown to be associated with SSC fate. Unique 3-gene identifiers distinguish 11 spermatogenic cell types in mice and humans	(100)
8	2018	Human	10 (2 Norma l men, 7 OA and 1 NOA)	Spermatogonia, meiotic germ cells, and haploid spermatids	Out of 3,243 testicular cells, 3,028 were filtered. Additionally, 2,854 cells with normal spermatogenesis and 174 cells from a non-obstructive azoospermic (NOA) donor were analyzed.	2 normal adults 30 & 60 years; 7 OA (27,29, 34,39,41,43,44 years) 1 NOA (24 years)	GSE106487	Modified Smart-seq2 followed by Illumina HiSeq 4000.	Total 7,378 expressed genes and 122,443 mRNA molecules in each individual cell are detected. Total 17 clusters have been reported out of which, 3 spermatogonia subtypes, 7 spermatocyte subtypes, and 4 spermatid subtypes. Stage dependent expressions of *Hmga1, Piwil4, Tex29, Scml1* and *Ccdc112* have been found with critical contribution of FGF and BMP pathways in SSC development.	(101)
9	2019	Human	4	Spermatogonial cells	33,585	2 neonatal (day 2 and day 7 of age) and 2 fertile adults aged 37 and 42 years)	GSE124263	10x Genomics followed by Illumina HiSeq4000	Neonatal and adult testicular “niche” cells and factors are defined. In adult testes, 4 clusters/ subsets of undifferentiated spermatogonia (SPG) with distinct markers [slow proliferative 2 populations of SPG -A (having *Utf1+* subset -1*, Nanos3+* subset - 2) and 2 populations mitotic SPG -B (*Nanos3*, *L1td1*, *Asb9, Dmrt1*, *Tuba3d*, *Dn mt1*, *Calr +* early diff-SPG B and *Sohlh2* + Diff-SPG)] have been identified.	(102)
10	2020	Human	4	Somatic and Germ cell and pubertal testis	19223(12854 filtered)	Juvenile (7-11 years) and pre-pubertal (13-14 years) testes. 2 Testoster one-suppress ed transgen der female testis (26,60 years)	GSE134144	10 x genomics followed by Illumina HiSeq2500 (Homo sapiens) Illumina NovaSeq 6000 (Homo sapiens)	This transcriptional atlas of the developing human testis provides major insights into developmental changes and key factors/pathways that accompany male puberty. Data show distinctive phases of germ cell differentiation, common progenitor for Leydig and peritubular myoid cells, two distinct transcriptional states of pre-pubertal Sertoli cells.	(103)
11	2020	Human/ Monkey/Mouse	4 Human; 5 Monkey	Somatic and Germ cells of testis	Human (13,837); Monkey (21,574)	20-40 years; 4-13 years	GSE142585	Drop-seq followed by Illumina HiSeq 2500 (Homo sapiens) Illumina HiSeq 2500 (Macaca mulatta)	Multi-species analyses reveal conserved and divergent testicular transcriptional dynamics. Xenotransplantation shows*Tspan33* as a marker of SSC, 6 different sub-populations of spermatogonial cells are defined based on specific markers. Phylogenetic differences in ligand-receptor interaction in testis are defined.	(104)
12	2020	Cynomolgus	4 (1 infant, 1 juveni le 2 pubertal)	Spermatogonial cells, meiotic germ cells	filtered data with 17,792 cells	1 year old infant, 2 year old juvenile, and 4 years old pubertal	E-MTAB-8979	10x Genomic followed by Illumina HiSeq 4000 or NovaSeq 6000 using paired- end sequencing	This data reveals critical markers outlining SSC differentiation (*Utf1, Uchl1* and *Dmrt1*). Genes associated with DNA damage response pathway has been examined throughout spermatogenic progression. Phylogenetic conserved features along with divergent multicopy and ampliconic gene content found in meiotic sex chromosome inactive (MSCI) for primates and rodents.	(105)
13	2020	Human	2	Somatic and germ cells of testis	853 PGCs, 2,854 normal testicular cells and 228 Sertoli cells	Male embryo and testis from (NOA) man	Unknown	Modified STRT-seq	All testicular cells express *ACE2* and Sertoli cells show highest expression level, with age *ACE2* expression gets decreased	(106)
14	2020	Human	29	Somatic and germ cell of pubertal testis	Spermatogo nial cells	fertile men aged between 30 and 50 y	SE144085	10 x Genomics	*Gdnf* and *BMP8b* broadly support long-term spermatogonial (*Spg*) culture, while activin A selectively promotes differentiated Kit+ *Spg* cells. Inhibition of AKT pathway specifically support primitive human undifferentiated *Spg*.	(107)
15	2020	Human and mice	02	Germ cells	2554	Adult men with OA 40 and 45 years of age		Drop microfluidics system flowed by Illumina NextSeq 500/550	Spermatogenic genes display lower rats of mutations with low diversity in the population. Genes, remain silent show diverge and rapid evolutionary rates.Germ;ine specific mutational signmature is generated by TCR following 3’-pyrimidine rule.	(108)
16	2020	Human, Mice	3	PGCs/ Gonocytes	16429	Fetal testes 17–18 weeks of gestation	GSE15 3819, GSE86146, GSE124263, GSE117101.	10x Genomics, followed by 2 × 150 paired- end sequencing on Illumina HiSeq 4000 or NovaSeq 6000	This study presents *in vitro* protocol for germ-line specification. The h-iPS generated PGC-like cells further get reconstituted as M-prospermatogonia-like cells and T1 prospermatogonia-like cells and closely resemble with human T1- prospermatogonia in vivo exhibiting diminished proliferation. Dynamic and stage-specific regulation of transposable elements during prospermatogonial specification have been found.	(109)
17	2021	Human	2	PGCs and somatic cells	∼32,500 cells	Fetal first trimester and neonatal 5 months old	GSE143356 GSE161617	10X genomics	A transcriptional cell atlas of the fetal and postnatal human testes. Fetal sematic cells originate from common progenitor by 7 weeks post fertilization and by 14 weeks, PGCs exit mitosis, downregulate pluripotent transcription factors, and strongly resemble the state 0 spermatogonia .	(110)
18	2021	Human, Mice	2	Spermatogonial cells	Slide-Seq	3-10 months in mice and 25 and 32 years for human	PRJNA668433	Slide-seq tools	Combining Slide-seq with targeted *in situ* RNA sequencing, this study demonstrates specific specific differences in the cellular compositions of spermatogonial microenvironment between mouse and human testes. Furthermore, In developing Gc *Habp4* (hyaluronan binding protein 4) has been identified as a potential regulator of chromatin remodelling	(111)
19	2022	Human Mouse	22	Human gonadal and adjacent extra-gonadal tissues (female *n* = 33, male *n* = 22) Mouse fetal gonads	347,709, 96,174 and 40,742 cells, 63,929 cells	First and second trimester s of gestation (6– 21 PCW) Embryon ic 10.5, 11.5 and 12.5 days	E- MTAB -10551	10× Genomics followed by Illumina HiSeq4000; Novaseq 6000	In both species, sex determination involves upregulated *Sry* and *sPax8s*, a gonadal lineage located at the gonadal–mesonephric interface. In human testes, *Siglec* *15* ^+^ and *Trem2^+^* fetal testicular macrophage populations are identified. This study provides a comprehensive spatiotemporal map of human and mouse gonadal differentiation, which can guide *ex vivo* spermatogenesis. They identified specific human regulatory programs governing the development of germ-line and somatic cell lineages, comparing these stages with equivalent ones in mice	(27)
20	2022	Human	9 male embryos/ fetuses and 6 female embryos/ fetuses	PGCs, gonocytes and fetal somatic cells	31006 for 10x 709 for modified STRT-seq	6 - 23 weeks of gestation	HRA000344	10× Genomics/mod ified STRT-seq Followed by Illumina HiSeq 4000	Clustering analyses of fetal gonadal tissues reveal several novel cell subsets, viz., *Pou5f1^+^Sparc^+^ FGCs* and *Krt19* ^+^ somatic cells. BMP signalling found to be critical in cell and stage-manner and promotes the *gonocyte-to-spermatogonium* transition. Biosynthesis of T also get transferred from fetal Sc to ALc.	(112)
21	2022	Human/ Mouse	12 (4 Young and /8 Old)	All testicular cells	44,657	(17-22 years); and (62-76 years)	GSE182786; PRJNA757777	10x Genomics, Followed by Illumina NovaSeq 6000	Age dependent spermatogenic dysregulation found to be more prominent as compare to that found in SSC. Altered pathways includes common inflammatory and hedgehog signalling selective metabolic signalling in Sc, T production in ALC, apoptosis in PTc etc . The extent of such dysregulation found to be associated with obesity in older but not in younger men.	(113)
22	2022	Monkey	3	Whole Testicular tissues	7,500 nuclei	Young (5-6 years); Old (18-21 years)	GSA (CRA007812)	10× Genomics followed by NovaSeq 6000 (Illumina, 20012866)	This work depicts in-depth transcriptomic traits of testicular aging at single-cell resolution, Sc found to be the most susceptible to aging, with significant decline in *Wt 1* expression.	(114)
23	2023	Human/ All primates/Mice	2 (Human), 3 (Chimpanze e), 2(Bon obo), 2 (Gorilla), 1 (Gibb on), 2 (maca que), 2 (marmoset)	Whole adult testes	97,521 single- nuclei	28, 32 years (Human) 16,21,45 years (Chimpanzee), 15,36 years (Bonobo), 43,51	s E- MTAB -11063; E- MTAB -11064	10x Genomics followed by NextSeq 550 (Illumina)	This study elucidates the phylogenetic analyses of spermatogenic transcriptional dynamics based on single-nucleus transcriptomic data obtained from testes across 11 mammalian species. Results indicate critical aspects of molecular evolution of spermatogenesis like conserved transcriptome differences between X- and Y-bearing spermatids and meiotic sex-chromosome inactivation (MSCI) process etc.	(115)
24	2023	Human/ Mouse	4 norma l and 9 NOA patients	Meiotic germ cells	Normal (1,097); NOA1 (176 cells) and NOA2 (130 cells)	unknown	GSE235324	Illumina HiSeq 4000 (humans); Illumina NovaSeq 6000 (mice)	This study provides a multi-omics-based spermatogenic landscape at single-cell resolution. Abnormal DNA hyper-methylation has been detected in leptotene spermatocytes of NOA patients. Functionally, functional interference in DNA demethylation affects meiotic recombination and fertility.	(116)
25	2023	Human	–	Parthenocytes (PGs) and Andandrocytes (AGs)	–	–	NA	HiSeq2500 and NovaSeq platforms	They showed transcriptomic profiles comparison between different stages of early development in AGs and PGs. PGs activate primitive genes until the 4-cell stage with increased methylation, challenging the belief that only early stages are totipotent and hinting at totipotency in cleavage-stage AGs, emphasizing paternal transcript significance.	(117)
26	2023	Baboon and Rhesus Macaqu es	2 baboo ns, 2 macaq ues	SSC	5000 cells	baboons (new- born and 26 months) macaque s (15 and 20 months)	GSE222105;	10x Genomics followed by Illumina HiSeq2500	Human spermatogonia form discrete groups, whereas baboon and rhesus spermatogonia are found to be less heterogeneous. A cross-species analysis reveals significant differences between primate and mouse SSCs. For example, genes coding for components and regulators of the actin cytoskeleton cortical for cell- adhesion are enriched in primate-specific SSC. In humans, both SSC and progenitor spermatogonia were found to be Adark, while Apale spermatogonia appears biased towards differentiation.	(118)
27	2023	Human/ Mouse	4	SSC	Not Applicable	7–18 weeks old mice and Human males 21- 48 years	PRJNA668433)	Slide-Seq	PTN and EPHA1 are potential SSC niche factors niche and spatial alteration in ligand-receptor paring leads to diabetes-induced infertility	(119)

**B:** Exclusively Rodents.

S.No	Year	Species	Sample Target Group or specific cell types	Number of cells examined	Age of the samples	GEO Accession Number	Platform	Major Findings and Critical Outcome	References
1	2018	Mouse/ Human	Adult spermatogon ia, spermatocyte s, spermatids, Steady-state Spermatogenic cells	62,000 cells	6-day postpartum (P6) and adult	GSE108970; GSE108977; GSE109033	Illumina NextSeq 500; Illumina HiSeq 3000	The Mammalian Spermatogenesis Single- Cell Transcriptome, from Spermatogonial Stem Cells to Spermatids.	(100)
2	2018	Mouse	2,500 cells from 8-week adult mice testis	2550 cells	2 adult (8 weeks) testis	GSE104556	Illumina HiSeq 2500	They covered all stages comprehensively, offering an ideal resource for marker discovery and analysis of differentiation processes. This dataset serves as a reference for future studies in disrupted spermatogenesis using single-cell RNA sequencing.	(120, 121)
3	2018	Mouse	Adult testis	35,000 cells	7,8,9,20 weeks	GSE112393	Illumina HiSeq 2500	They utilize single-cell RNA sequencing to create a resource on mouse spermatogenesis which aims to unravel the diverse cell types within the adult testis, pinpoint factors influencing germ cell differentiation, and uncover various types of somatic cells involved in this process.	(122)
4	2018	Mouse	Embryonic stage somatic cells	435 cells	Embryo day E10.5, E11.5, E12.5, E13.5, E16.5	GSE97519	Illumina HiSeq2000	They have identified a progenitor cell population expressing single Nr5a1 before sex determination. These cells undergo temporal fate specification, exhibiting competence windows to differentiate initially into Sertoli cells or later into fetal Leydig cells.	(123)
5	2019	Mouse	Spermatogonia and Spermatocyte	–	Postnatal (6d, 14d, 18d, 25d, 30d); Adult (8 weeks)	GSE121904	Illumina NextSeq; 500	Dynamic transcriptome profiles within spermatogonial and spermatocyte populations during postnatal testis maturation revealed by single-cell sequencing	(124)
6	2020	Mouse	Germ and somatic cell development during the perinatal period	8,916 cells	Embryonic stage 18.5, P2, P7	GSE130593	Illumina HiSeq 4000	This study pinpoints specific signaling pathways between somatic and germ cells during the perinatal phase, serving as a valuable resource for understanding testicular cell development during this time.	(125)
7	2020	Mouse	3-week mice injected with busulfan and rescued with AOS	27,000 cells	Treatment 3 weeks of age to 8 weeks of age	GSE131629	Illumina HiSeq 2000	They concluded that alginate oligosaccharides improved blood and testis metabolomes as well as the gut microbiota to support the recovery of spermatogenesis	(126)
8	2020	Murine	ECs from 11 mouse tissues including testis	>32,000 single Endothelia l Cells (ECs)	8-weeks male mice	Array Express E-MTAB- 8077	Illumina HiSeq 4000	Reported is a comprehensive murine atlas consisting of over 32,000 single ECs transcriptomes obtained from 11 mouse tissues including testis. Within these subclusters, diverse classical and tissue- specialized endothelial cell subtypes have been delineated.	(127)
9	2021	Mouse/ Human	–	–	3 and 10 months testis; Two healthy men (25 and 32 years)	PRJNA668433	Nextera XT; NextSeq 500/550 High and Mid Output Kit v2.5 (75 Cycles)	They generate a spatial transcriptome atlas of the mammalian testis, which is used to reveal the spatial organization of the testicular microenvironment and profile its changes under diabetic conditions.	(111)
10	2021	Mouse	PGCs	14,750	E10.5, W12.5, and E16.5	GSE136220	Illumina HiSeq 4000 (Mus musculus)	This study provides a molecular roadmap of GC sex determination at single-cell resolution and will serve as a valuable resource for future studies of gonad development, function and disease	(128)
11	2021	Mouse	cultured THY-1+ GCs w/wo FGF9	4,143 control and 4,482 FGF9-treated cells	–	–	NextSeq 500 (Illumina)	They concluded that FGF9 is an important regulator of SSC proliferation, operating through p38 MAPK phosphorylation and upregulating Etv5 and Bcl6b in turn.	(129)
12	2022	Mouse	Young and Old	37,571 cells	2 and 24 months	PRJCA00856	10X Genomics Chromium; NovaSeq 6000	This study reveals the first detailed map of aging in mouse testes at the single-cell level. It identifies changes in gene profiles, disruptions in stem cell balance, and the emergence of specific aging-related cell types, like macrophages. The findings offer a vast resource for studying age-related subfertility and suggest potential paths for diagnostics and targeted treatments against testicular aging by focusing on oxidative stress and inflammation.	(130)
13	2022	Rat	Adult LCs before and after EDS treatment	10,000	12 weeks	PRJCA006440	Illumina NovaSeq 6000	Identification of Rat Testicular Leydig Precursor Cells by Single-Cell-RNA- Sequence Analysis	(85)
14	2022	Rat	Testes from 3 animals were collected at each of 1, 3, and 7 weeks post-EDS treatment	4,000	12 weeks	PRJCA006139	Illumina HiSeq PE150	The study explored the effects of the androgen environment on the regulation of spermatogenesis. As this is the first single- cell RNA-Seq dataset for rat testes, it can also serve as a reference for future studies	(131)
15	2022	Mouse	WT and Alkbh5 KO testis	WT- 5,596 cells; KO- 6,816 cells	12 week	GSE190396	HiSeq X Ten (Mus musculus)	This study presents the inaugural single-cell RNA sequencing profile of ALKBH5- deficient mice's testes. It underscores ALKBH5's significance in germ cell development and spermatogenesis, providing fresh molecular insights. These discoveries might form the groundwork for further investigations into the origins and therapies for male infertility.	(132)
16	2023	Mouse	Control and Sertoli cell specific Scf- cKO mice testis	–	8-week-old	GSE161040	HiSeq X Ten (Mus musculus)	Here authors utilised fluorescent reporter mice and revealed widespread expression of the stem cell factor (Scf) across different testicular stromal cells, boosting Scf specifically in Sertoli cells significantly improved spermatogenesis, underscoring their crucial role in regulating this process.	(133)
17	2023	Mouse	fetal to neonatal transition	25,613	E18.5; Postnatal (day1; day3; day6)	GSE164439	Illumina HiSeq 2000 (Mus musculus)	The single-cell chromatin accessibility landscape in mouse perinatal testis development	(134)
18	2023	Mouse/H uman	Adult male mice; Mouse SSCs; 4 healthy men; Human SSCs	–	(7–18 weeks old)	PRJNA668433	Illumina NovaSeq S2 flow cell 100 cycle kit	Their findings suggested that inferring ligand-receptor (LR) pairs within the spermatogonial stem cell niche identified PTN and EPHA1 as potential factors within this niche. They also indicated that the spatial changes in LR pair expression are the root cause of infertility induced by diabetes	(118)
19	2023	Mouse	Adult WT, GRTH- Knockout, and GRTH knock-in mutant mice	7,000 cell for each sample	8 weeks old	GSE221226	Illumina NovaSeq 6000	These studies highlight the significance of pGRTH in acrosome biogenesis and the progression of round spermatids RS into ES during spermiogenesis.	(135)
20	2023	Mouse/H uman	WT mice treated with busulfan	10,000 cells	8 weeks old	GSE164787 Reanalysed GSE124263	Illumina NovaSeq 6000	This study provides a blueprint for understanding the development of the male germline and supporting somatic cells in humans. The germ cell subset markers identified are candidates to be used for clinical applications, including SSC therapy for treating infertility.	(136)
21	2023	Mouse	adult Ddx43+/+, Ddx43KI/KI and Ddx43– /–testes	17,133	adult	PRJNA650016; PRJNA838233	Illumina NovaSeq 6000	Their identification of RNA helicase DDX43 as a crucial regulator of chromatin remodeling in spermiogenesis showcases its pivotal role in this process. These findings underscore the significance of DDX43 in spermiogenesis and emphasize the efficacy of the single-cell-based approach in delineating cell-state-specific control in male germline development.	(137)
22	2023	Mouse	control and Kdm6a conditional knockout (“cKO”) Whole testis	WT- 19,378 cells; KO- 16,740 cells	–	GSE215112	Illumina NovaSeq 6000	The histone demethylase KDM6A, exhibits transient expression just before and during meiotic entry in spermatogenesis, playing a crucial role in maintaining epigenetic states across generations in the male germline.	(138)

**Table 4:** List of Human Participants Exhibiting Spermatogenic Dysfunction or Infertility: A Detailed Examination.

S.No	Year	Population	Number of Donar(s)	Age	Pathology/ Etiology reported	Number of Cells examined	GEO Accession Number	Platform	Major Findings/ Critical Outcome	References
1	2018	Chinese	2 Healthy donors, 7 Obstructiv e azoosperm ia (OA) donors, and 1 Non- obstructiv e azoosperm ia (NOA) donor	Healthy (30, 60 years); OA (39, 43, 27, 34, 44, 41, 29); and NOA (24 years)	SCOS found in one NOA donor	2854 (normal) 174 (NOA)	GSE106487	Modified Smart- seq2 followed by Illumina HiSeq 4000	Enrichment of γH2AX signal in NOA somatic cells, suggests their activation of DNA damage response mechanisms.	(101)
2	2019	German	1 donor	Unknown	47,XXY Klinefelter Syndrome (KS)	3289	GSE130151	10x Genomic s; Illumina NovaSeq 6000	On the transcriptional level, Klinefelter Gc (both spermatogonia and sperm) exhibit normal DNA methylation, however, Klinefelter patients show variations in the DNA methylation of imprinted regions.	(139)
3	2020	Danish	1 KS donor	Unknown	KS	3,289	Reanalyzed GSE130151	Illumina NovaSeq6000	KS likely causes early testicular damage, leading to fewer Gc, loss in Sc, and ALc changes. Genetic studies pinpoint specific gene alterations, especially in X-escapee genes, driving these issues. Sc dysfunction is emphasized as pivotal in Gc loss, urging wider research across ages and more KS patients to understand this condition better.	(140)
4	2020	Chinese	4 (2 OA and 2NOA)	Unknown	NOA	1212	OEP000778	Singleron GEXSC OPE foll owed by Illumina HiSeq X Ten	Transcriptional difference in OA and NOA, Sc-Sc junction genes *Syne2, Atp2b1, Mtdh* and elevated *Fate1*.	(141)
5	2020	Chinese	10 Healthy donors and 7 NOA donor	Infant to Adult	Testicular torsion , benign testicle mass , or contralateral testis to cryptorchidism NOA (SCOS)	88,723	GSE149512	10X Genomic s followed by Illumina NovaSeq 6000	This study has compared testicular cells (from 10 normal and 7 NOA) and found prominent maturational defects. Blocking *Wnt* signaling helps immature cells to support germ cell survival, offering new diagnostic /treatment strategies for NOA.	(64)
6	2021	Chinese	7 OA, 1 NOA and 2 normal persons	34, 36, 50 years	Hypospermatoge nesis	480	GSE157421	(STRT- seq) follo wed by Illumina HiSeq XTEN	Changes in autophagy-related genes, such as the upregulation of *Sqstm1* and the down-regulation of *Lc3a* in spermatids, were identified in NOA Gc , while *Cst3-* mediated autophagy potentially aids in maintaining SSC through *Oct4, Id1*, and *Nanos3*; additionally, inhibition of *Cst3* disrupted meiosis and spermatid formation.	(142)
7	2021	Italian	3 iNOA donors and 1 OA donor	iNOA (32/41/37 years) and OA (37 years)	idiopathic SCOS/ germ cell aplasia (OA and iNOA)	3880	GSE154535	10X Genomic s followed by Illumina NextSeq5 00/Nova Seq 6000	idiopathic Gc aplasia viz., immaturity of ALc, chronic tissue inflammation, fibrosis, and senescence phenotype of the testicular somatic cells, ratio of Lamin A/C transcripts and an active DNA damage response in Lc and PTc.	(143)
8	2021	German	3 healthy and 3 cryptozoo spermic donor	31, 33, 59 years healthy and 36, 39, 25 years Diseased	cryptozoospermi a	30,000	GSE153947	10X Genomic s followed by Illumina NovaSeq 6000	Alterations in the crypto-spermatogonial compartment having more of Piwil4^+^ undifferentiated spermatogonia, prolonged expression of *Egr4* and fewer SSC/ Adark spermatogonia. .	(144)
9	2021	Americ an	12 donors	Mix age	KS and idiopathic azoospermic (iNOA) male infertility	26300	GSE169062	10x Genomic s followed by Illumina HiSeq 2500	This study identifies a subpopulation of Sc (within multiple individuals having KS) lacking transcription from the XIST locus resulting elevated X-linked gene expressions. Furthermore, 72 pathways upregulated in KS suggesting changes in interstitial cells due to loss of X inactivation in Sc.	(145)
10	2022	Mix	–	-Do-	NOA	–	GSE1495 12; GSM4504195; GSM 4504196; GSM450 4197; GS M450419 5; GSM45 04196; GSM450 4197 (Data Reanalyzed)	–	Elevated ALc and macrophages in iNOA patients testes Lc specific markers *Lhx9*, *Klf8*, *Klf4*, *Arid5b* and *Rxrg*) in NOA, and macrophages-specific TFs (such as *Pou2f2*, *Spib*, *Irf5*, *Cebpa*, *Elk4* and *Klf6*) in NOA,	(146)

## 6 New progress

This comprehensive review of literature on testicular Sc-RNA-seq highlights the complexity of testicular tissue composition and the uniqueness of cellular transcriptional dynamics. The cutting-edge approach has unveiled a heterogeneous landscape within the testis, providing insights into spermatogenic Gc, somatic PTc and interstitial Lc and supportive Sc associated with SSC microenvironments. Studies employing Sc-RNA-seq have examined the transcriptional profiles of various Gc populations at distinct developmental stages elucidating the molecular regulation of spermatogonial differentiation, meiosis, and sperm maturation. The application of Sc-RNA-seq has not only enriched our understanding of testicular cell heterogeneity but has also identified novel markers and regulatory pathways critical for male infertility and reproductive health (147). Integration of multi-omics data and advanced *in-silico* analyses offers a comprehensive view of the testicular transcriptomic landscape, fostering discoveries with profound implications for both basic biology and clinical applications in reproductive medicine.

A significant development in the field of testicular transcriptomics requires an inclusive assimilation/integration of data obtained from Sc-RNA-seq analyses with spatial transcriptomic information (32). For example, Chen and colleagues employed the “spatial transcriptomic analysis” technology, creating a spatial atlas that delineates testicular gene expression at near-single-cell resolution in the human testis (111). Similarly, Garcia-Alonso et al. conducted integrated analyses of male gonadal development by fusing Sc-RNA-seq and spatial transcriptomics, offering a more comprehensive and in-depth perspective (27).

## 7 Current challenges

**Figure 9:**
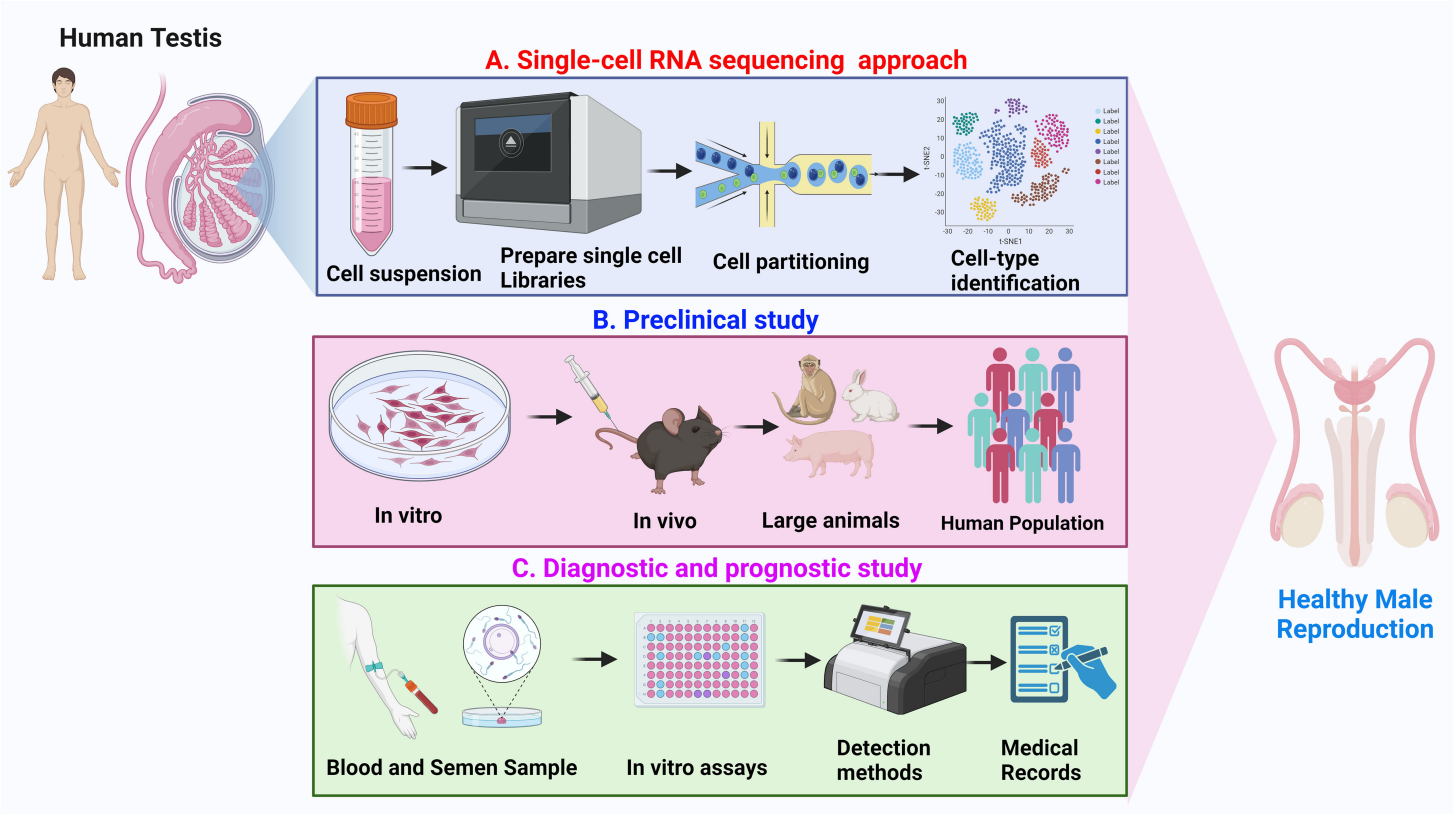
Overview of application of Sc-RNA-seq data. **(A)** Single-cell RNA sequencing approach: Depicts the workflow from human testicular tissue sample to single-cell suspension, followed by library preparation for RNA sequencing, cell partitioning, and subsequent cell-type identification using clustering analysis. **(B)** Preclinical study: Represents the preclinical research progression, beginning with *in vitro* experiments, advancing through *in vivo* studies in rodents and large animals, and culminating in analysis within a human population. **(C)** Diagnostic and prognostic study: Illustrates the process of collecting blood and semen samples, performing *in vitro* assays, utilizing various detection methods, and integrating findings with medical records for the advancement of healthy male reproduction diagnostics and prognostics.

The integration of these methodologies is anticipated to augment future investigations on the human testis and male infertility, affording a comprehensive understanding of functional/impaired spermatogenesis. Although Sc-RNA-seq analyses have corroborated the notable presence of immune cells (e.g., macrophages - both M1 and M2, T cells, mast cells, and B cells) in the testicular microenvironment, the functional roles (and also transcriptional patterns) of these cells in regulating male infertility development remain elusive (85). However, despite the current high cost of Sc-RNA-seq, several of the studies sincerely lack a direct clinical orientation Therefore, diverse testicular pathologies, viz., cryptorchidism, AZFc deletions, azoospermia post chemo/radiotherapy, and teratospermia, demand focused exploration of Sc-RNA-seq analyses. The ongoing refinement and cost mitigation of this technology holds future promise for clinical applications, such as diagnostics, classification, prediction of sperm retrieval, and evaluation of hormonal treatment efficacy. **Figure 9** illustrates the probable applications of Sc-RNA-Seq data towards framing appropriate preclinical studies (both *in vitro* and *in vivo* approach) leading to potential future diagnostic (assays/tools/kits) in male reproductive health care.

## 8 Concluding remarks and future directions

Exploring the intricate landscape of testicular function and pathology requires a comprehensive understanding spanning from the fundamental processes of development to the complexities of the disorders. This review has examined the uncharted territory of the testicular single-cell transcriptional atlas, illuminating the diverse cellular populations and their orchestrated molecular dynamics. From the genesis of Gc to the intricate orchestration of somatic cell interactions, this exploration navigates the transcriptional intricacies shaping testicular development. Furthermore, it delves into the disruptive signatures underlying various testicular disorders, shedding light on potential avenues for diagnostic and therapeutic advancements. By unraveling the cellular intricacies from a single-cell perspective, this review aims to provide comprehensive insights bridging the developmental trajectory to the manifestations of disorders within the testicular microenvironment. We also summarize the previous attempts deploying Sc-RNA-Seq on clinical testicular biopsies (**Table 4**) that have significantly enriched our understanding of testicular physiology e.g.- regulation of SSC micro-environment and testicular somatic cell maturation, etc. Finally, the increasing availability of publicly accessible testicular Sc-RNA-seq datasets provides researchers with greater possibilities for conducting personalized data analyses to address diverse research requirements. We anticipate that this review will not only facilitate the comprehensive understanding of spermatogenic transcription during normal and impaired (sub-fertile/infertile) conditions but also serve as a valuable reference for critical diagnosis and cure for some forms of unexplained/idiopathic male infertility.

## Author contributions

MT: Conceptualization, Data curation, Formal analysis, Investigation, Methodology, Software, Writing – review & editing. IB: Visualization, Formal Analysis, Writing – original draft, Writing – review & editing. MM: Data curation, Formal analysis, Writing – review & editing. VB: Data curation, Formal analysis, Writing – review & editing. MC: Conceptualization, Data curation, Investigation, Supervision, Validation, Visualization, Writing – original draft, Writing – review & editing.
