# Supplementary material for: Understanding testicular single cell transcriptional atlas: from developmental complications to male infertility
Source: Front Endocrinol (Lausanne). 2024 Jul 11;15:1394812. doi: 10.3389/fendo.2024.1394812 (PMC11269108; doi:10.3389/fendo.2024.1394812)
Supplement: Supplementary file 1 [file DataSheet_1.docx]

# Supplemental Table 1: Summary of male infertility

# Supplemental Table 1A:

# Clinical details of Semen Abnormalities.

This table categorizes various conditions related to semen quality and sperm characteristics. It describes conditions such as Aspermia (no semen/ejaculation), Azoospermia (complete absence of spermatozoa), Oligozoospermia (less than 10 million sperm/ml of semen), and more, along with descriptions of each condition. These conditions range from the absence or low count of spermatozoa to issues with sperm motility and morphology, indicating the diverse clinical presentations of male infertility [Adopted from Agarwal et al., 2021 (3)].

| **Name** | **Description** |
| --- | --- |
| Aspermia | No semen/ejaculation |
| Azoospermia | Complete absence of spermatozoa |
| Oligozoospermia | <10 million sperm/ml of semen |
| Necrozoospermia | Nonviable immotile sperm in semen |
| Oligoasthenozoospermia | Motile density <8 million sperm/ml |
| Asthenozoospermia | Around 50 % of sperm with low progressive motility |
| Teratozoospermia | >50 % of sperm with abnormal morphology |
| Oligoteratozoospermia | < 50 % of sperm with abnormal morphology |
| Asthenoteratozoospermia | Around 50 % of sperm with low progressive motility and impaired morphology |
| Globozoospermia | Sperm appear to have large round heads without an appropriately developed acrosome due to deletions/mutations in *Dyp19l2* or *Spata16* or *Pack1* |
| Hematospermia | Presence of erythrocytes ( RBCs) in semen |
| Leukospermia / Pyospermia | Presence of leucocytes (WBCs) in semen above the threshold limit |
| Polyzoospermia | Excessively high sperm concentration in semen |
| Normozoospermia | Total morphologically normal and progressively motile sperm concentration equal or above the lower (10 million sperm/ml) limit. |

**Supplemental Table 1B:**

**Etiologies and Frequencies of Male Infertility.**

Outlines the genetic and environmental factors contributing to male infertility. It lists specific abnormalities, their effects on sperm quality, and the frequency of these conditions in the affected population. For example, Chromosomal aberrations like Klinefelter’s syndrome, Y chromosomal micro-deletions, and gene mutations such as CFTR, and the impact of lifestyle and environmental factors on sperm quality are included, providing a comprehensive overview of the multifactorial etiology of male infertility [Adopted from Agarwal et al., 2021 (3)].

| **Sl.**  **No.** | **Abnormality/ Etiologies** | **Effect/ Symptoms** | **Approximate Frequency (%)** |
| --- | --- | --- | --- |
| **1.** | **Chromosomal aberrations** Klinefelter’s syndrome 47, XXY, variance (like 9qh+ heteromorphism), Other sex chromosome alterations  Robertsonian/ Reciprocal translocations | Azoospermia to severe oligospermia | 5–10  0.1–0.2  0.5–1 |
| **2.** | **Y chromosomal micro-deletions**  AZFa ,  AZFb,  AZFc,  AZFb + c  Partial AZFc deletion | Azoospermia (SCOS)  to severe oligozoospermia | 0.5–1  do  3–7  0.5–1  3–5 |
| **3.** | **Gene mutation**  Cystic fibrosis transmembrane conductance regulator (*Cftr*)  Androgen Receptor (*Ar*) | Obstructive azoospermia  to | 60–70  2–3 |

|  | Insulin-like 3 (Insl3)  and  *Lgr8* | Oligozoospermia Cryptorchidism | 4–5 |
| --- | --- | --- | --- |
| **4.** | **Hypogonadotropic hypogonadism**  Congenital: Kallmann syndrome Acquired: tumor, infection, autoimmune, pituitary infarction, and drug use | Azoospermia to  oligozoospermia | 0.5  0.25 |
| **5.** | **Anatomical Deformities**  Anosmia  Cryptorchidism  Congenital absence of vas deferens Varicocele | Azoospermia to severe oligozoospermia | 1-2.5 |
| **6.** | **Acquired /Life style/Environmental factors**  Infection or Systemic diseases like Prostatitis, prostatovesciculitis , epididymitis, mumps orchitis, liver cirrhosis, renal failure  Chemotherapy, medications, radiation, heat Urogenital tract obstruction  Sexual dysfunction (erectile or ejaculatory dysfunction)  Smoking/Alcoholism/Drugs/Medications/Surgery  Exposure to Endocrine disruptors/pollutants/toxicants  Obesity Psychological stress Advanced paternal age | Azoospermia to severe oligospermia | 15-20 |
| **7.** | **Idiopathic** | Azoospermia to severe oligozoospermia | 20-30 |

**Supplemental Table 1C:**

**Major Histo-pathological Features Found in Non- obstructive azoospermia (NOA)/Primary Testicular Failure.**

Presents the major causes and their effects on the histopathology observed in non- obstructive azoospermia (NOA) or primary testicular failure. It includes conditions like Germ cell (Gc) aplasia or Sertoli cell-only syndrome (SCOS), hypospermatogenesis, and spermatogenic arrest, along with the causes ranging from genetic mutations to environmental factors. The table describes the pathological impact of these conditions on spermatogenesis, such as the reduction in the diameter of seminiferous tubules or the interruption of germ cell differentiation at various stages (21).

| **Name** | **Major Cause** | **Effect/ Etiologies** |
| --- | --- | --- |
| 1. Gc aplasia or ***Sertoli cell only*** syndrome ***(SCOS)*** | Undescended testes, Y chromosome micro- deletions,  anti-neoplastic chemotherapy with radiation and  viral infections like mumps orchitis. | Reduction in diameter of the seminiferous tubules containing only Sc without any Gc leading to non-obstructive azoospermia (NOA). In congenital forms PGCs fail to migrate from the yolk sac and undergo massive apoptosis. |
| 2. Hypo- spermatogenesis | Y chromosome microdeletions, genetic mutation, acquired or idiopathic | Moderate or severe reduction in the number of differentiating Gc including mature spermatids in the tubules with a lower testicular volume/size. FSH is elevated with low Inhibin B. |
| 3. Spermatogenic  Arrest | Trisomy, balanced autosomal anomalies (translocations, inversions) or deletions in the Y chromosome (Yq11). | This is the maturational interruption of normal Gc differentiation at multiple stages e.g.- spermatogonial  / spermatocyte or at spermatid. |
